# Chemical Components and Biological Activities of the Genus *Phyllanthus*: A Review of the Recent Literature

**DOI:** 10.3390/molecules23102567

**Published:** 2018-10-08

**Authors:** Muhammad Farrukh Nisar, Junwei He, Arsalan Ahmed, Youxin Yang, Mingxi Li, Chunpeng Wan

**Affiliations:** 1 Interdisciplinary Research Centre in Biomedical Materials (IRCBM), COMSATS University Islamabad, Lahore Campus, Lahore 54000, Pakistan; farrukh.nisar@cuilahore.edu.pk (M.F.N.); arsalanahmed@cuilahore.edu.pk (A.A.); 2 Research Center of Natural Resources of Chinese Medicinal Materials and Ethnic Medicine, Jiangxi University of Traditional Chinese Medicine, Nanchang 330004, China; hjwjn2008@163.com; 3 Jiangxi Key Laboratory for Postharvest Technology and Nondestructive Testing of Fruits & Vegetables, Collaborative Innovation Center of Post-Harvest Key Technology and Quality Safety of Fruits and Vegetables, College of Agronomy, Jiangxi Agricultural University, Nanchang 330045, China; yangyouxin@jxau.edu.cn (Y.Y.); liming.xi@hotmail.com (M.L.)

**Keywords:** *Phyllanthus*, traditional medicines, phytochemicals, biological activities

## Abstract

Medicinal plants have served humans since prehistoric times to treat various ailments. Both developed and underdeveloped countries rely on traditional systems of medication using natural sources from plants. *Phyllanthus* is one of the largest genus in the family Phyllanthaceae, comprising over 700 well known species cosmopolitan in distribution mainly in the tropics and subtropics. *Phyllanthus* species are being in constant used in traditional medications to cure an array of human diseases (constipation, inhalation related, arthritis, loss of appetite, injuries, conjunctivitis, diarrhoea, running nose, common cold, malaria, blennorrhagia, colic, diabetes mellitus, dysentery, indigestion, fever, gout, gonorrheal diseases of males and females, skin itching, jaundice, hepatic disorders, leucorrhea, vaginitis, menstrual irregularities, obesity, stomach pains, and tumors), confectionaries, food industry, and in some pesticides. *Phyllanthus* species are rich in diversity of phytochemicals e.g., tannins, terpenes, alkaloids, glycosidic compounds, saponins, and flavones etc. More in depth studies are a direly needed to identify more compounds with specific cellular functions to treat various ailments.

## 1. Introduction

The utilization of herbal plants to treat various human diseases is cosmopolitan and universal, particularly in third world countries due to their easy access and low cost, compared with advanced Western medicines [1,2]. Plants with medicinal properties are efficiently used, mainly by the rural and/or tribal communities in remote areas, not only today but also in ancient communities in prehistoric times [2,3,4], and are highly respected in certain tribal communities due to myths such as being a tonic of life [5]. These herbal pharmacological formulations were routinely used throughout human history without any scientific evidence, and now are being used in practical clinical trials to find their optimum and beneficial therapeutic dose ranges. This use has led to the development of a huge number of advanced Western medicines such as the cardiotonic steroid digitoxin and anticancer drugs like vincristine and vinblastine [1]. Many drugs such as praziquantel, well known for its resistance and insensitivity of juvenile schistosomes, have helped researchers focus on the exploration of more medicinal flora for novel alternative drug resources [6].

*Phyllanthus* is one of the largest genus in the family Phyllanthaceae, with 11 sub-genus that comprise over 700 well known species and are cosmopolitan in distribution, mainly in the tropics and subtropics [7]. Most important species traditionally used for treatment of various human ailments belonging to the sub-genuses Cicca, Kirganelia and *Phyllanthus*. Plants of the genus *Phyllanthus* were utilized as herbal formulations for centuries in many Southeast Asian countries, Brazil, India and China [7]. The extracts of *Phyllanthus amarus* (*P. amarus*) show good antioxidant activity, along with antibacterial potential, particularly in conditions including diarrhoea, dysentery, dropsy, running nose, winter common colds, blennorrhagia, colic, indigestion, alternating fevers, hepatitis, and malaria [8,9]. *Phyllanthus niruri* L. (*P. niruri*) has been extensively reported in traditional and folk medication systems to treat various diseases including asthma, joint pains, loss of appetite, constipation, injuries, corneal opacity, conjunctivitis, diabetes mellitus, dropsy, gout, gonorrheal dieases of males and females, inflammatory diseases, skin itching, hepatic disorders, kidney stones or failures, leucorrhea, vaginitis, menorrhagia, irregularities in menstrual cycles in females, obesity, scabies, stomach pains, tumors, typhoid fever, urinogenital disorders viral infections and many more [10]. The potential of the extracted active phytochemicals and, therefore, the biological actions of the extracts from *P. amarus* are said to be highly altered by the solvents and the methods of extraction used [11]. Different parts of *Phyllanthus* species plants have different pharmacological activities thought to be due to the specificity of bioactive molecules in specific parts of the plant. For instance, the fruit of *P. emblica* is a rich source of vitamin C along with various bioactive ingredients [12]. *P. emblica* L. fruit is the most studied part of this plant, which has disclosed a huge number of bioactive compounds displaying diverse biological activities [13]. Moreover, *P. emblica* is important for its commercial use in the bpreparation of wines. The utilization of *P. muellerianus* in traditional medical treatments to cure several ailments, including fevers, toothache, dysmenorrhea, anemia, and paralysis has been reported [14]. Medicinal flora have generated a significantly huge number of potent, crucial and important phytochemicals as an alternative to allopathic medicines and are regarded as equivalent in the formulation and design of new drugs [15].

Plants of the genus *Phyllanthus* are widely used in traditional medicine and known as natural sources of antioxidant compounds. Recent investigations support their genoprotective activity against physico-chemical mutagens such as ultraviolet radiation (UVR) [16]. The fruits of *P. emblica* L. are extensively used as a functional food as well as a traditional medical remedy in India, China, and adjacent areas because of its amazing nutritional and pharmacological potential [17]. Pesticides comprise a diverse class of chemical compounds applied to kill or prevent fungal growth, insects, weeds and repel many soil rodents. Moreover, these chemicals are highly beneficial because they help to safeguard forests and farm crops against the losses posed by various crop pathogens, hence aiding to increase overall yield of food production. These phytochemicals efficiently control various insect-vector borne diseases of different crop plants, but their increased utilization poses serious threats to human health, erases bio-diversity at the local scale along with irrepairable environmental change and damage to natural ecological habitats [18]. *P. emblica* L. fruit extract has diverse uses in confectionaries, the food industry, and as a traditional medicine [19]. Many studies have evidenced that it contains various potential and active phytochemicals are can be used for different human diseases linked with human lifestyles when mainly taken up as neutraceuticals [20].

*Phyllanthus* species have been well studied in recent decades, but while most of the individual studies have focused in the composition of the constituents in various plant parts and the biological activities of plant extracts, hardly any study exists which simultaneously describes the biological roles of the various constituents of *Phyllanthus* species. Hence, this review aims to summarize the most recently published data (2016–2018) concerning the biological activities of various potent constituents found in *Phyllanthus* plant extracts.

## 2. Chemical Components

**Figure 1:**
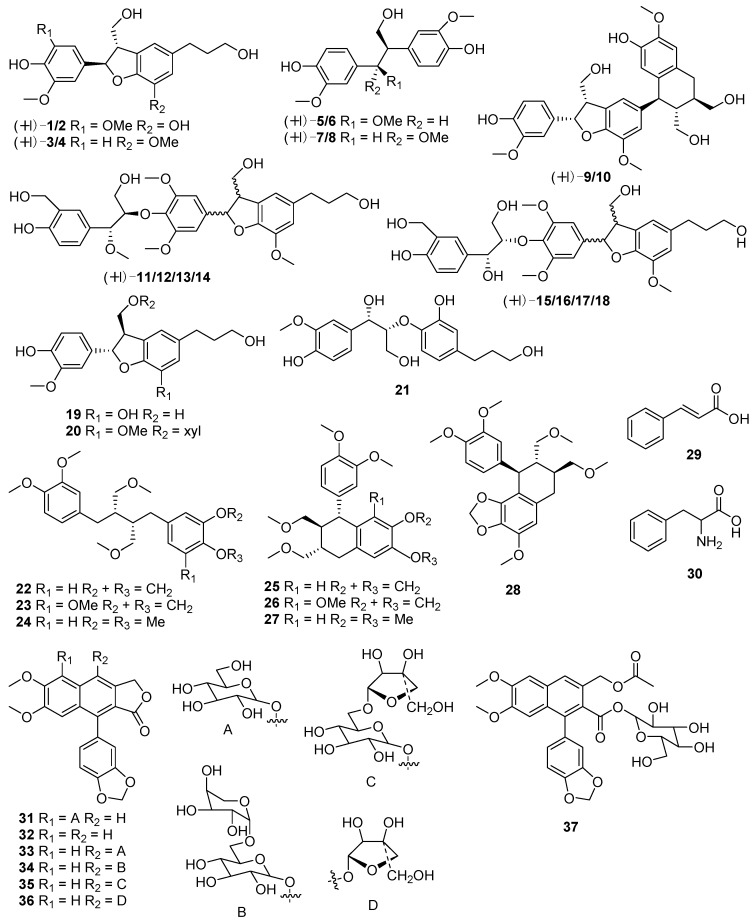
Phenylpropanoids from various *Phyllanthus* species.

**Figure 2:**
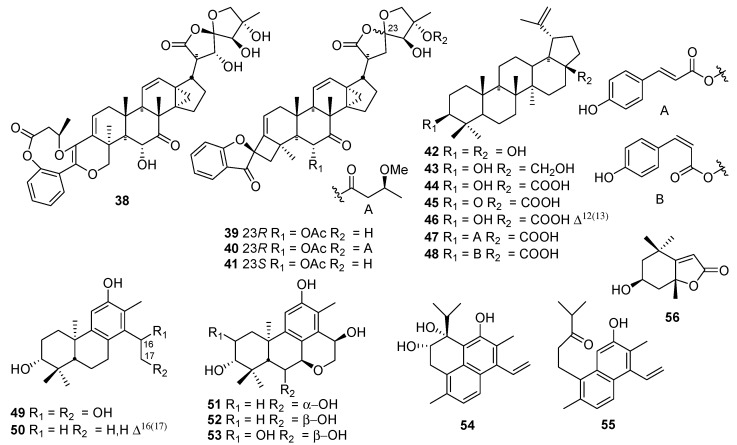
Terpenoids from various *Phyllanthus* species.

**Figure 3:**
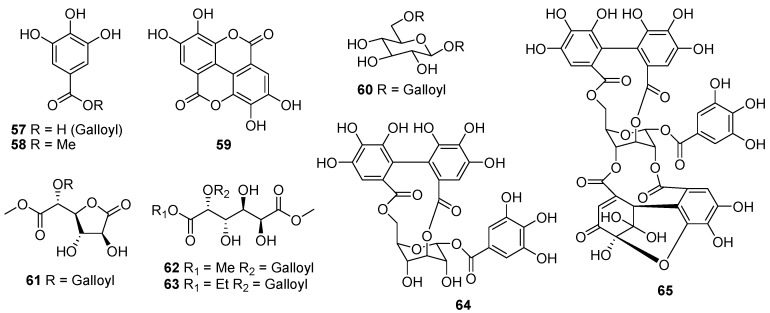
Phenolic compounds from various *Phyllanthus* species.

**Figure 4:**
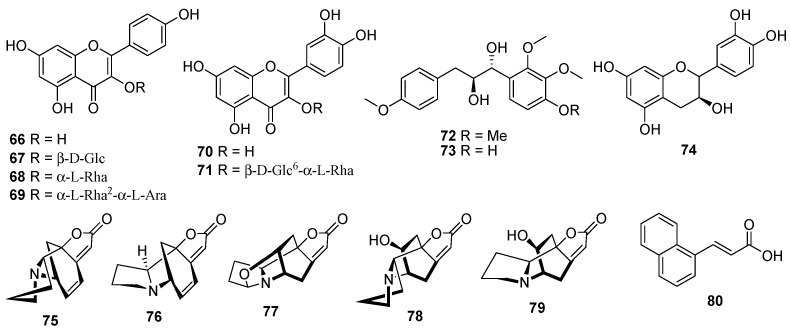
Flavonoids, alkaloids and other compounds from various *Phyllanthus* species.

An initial phytochemical exploration of *Phyllanthus* species reported the occurance of terpenoids, alkaloids, glycosides, flavonoids, tannins, and saponins [7,21]. Phenolic compounds, especially tannins, are the major constituents of *Phyllanthus* plants. More than 100 phenolic constituents with diverse biological activities were comprehensively identified in the fruits of *P. emblica* L. using HPLC-MS [22]. It is also emphasized that different parts of *Phyllanthus* plants have different isomers of the same compounds. *Phyllanthus* species are rich in phytochemical diversity, with compounds such as tannins, phenylpropanoids, terpenoids, phenolic compounds, flavonoids, alkaloids, saponins and many of their glycosides. Almost 81 compounds have been isolated from *Phyllanthus* spp. during 2016–2018, the majority of which were phenylpropanoids, triterpenoids, diterpenoids, and flavonoids. The components isolated from each *Phyllanthus* species are summarized in the current review (Figure 1, Figure 2, Figure 3 and Figure 4).

### 2.1. Phenylpropanoids

Phenylpropanoids constitute the most prevalent class of compounds in the genus *Phyllanthus*. Thirty seven compounds (Figure 1, Table 1) were identified from *P. glaucus* [23], *P. amarus* [24,25,26], *P. urinaria* [24] and *P. brasiliensis* [27]. Among these compounds lignans such as neolignan, norlignan and sesquineolignan were the most prominent. Interestingly, most of the compounds were present in the form of enantiomers in *P. glaucus*, including nine pairs of enantiomeric lignans **1**–**18**.

### 2.2. Terpenoids

Terpenoids are another major class of chemicals in the genus *Phyllanthus*. About 19 compounds (Figure 2, Table 2) including 11 triterpenoids **38**–**48**), seven diterpenoids **49**–**55**, and one monoterpene **56**) were identified in *P. hainanensis* [28], *P*. *urinaria* [29,30], and *P. acidus* L. [31,32]. It is noteworthy that compound **38** is a new skeleton compound, which incorporates an unprecedented 6/9/6 heterotricyclic system in the lower-left and a highly oxygenated 5,5-spirocyclic ketal lactone motif in the upper-right. Compounds **42**–**48** are lupine type pentacyclic triterpenoids.

### 2.3. Phenolic Compounds

Seven phenolic compounds, including one new mucic acid 1-ethyl 6-methyl ester 2-*O*-gallate (**57**), together with six known phenolic compounds such as gallic acid (**58**), methyl gallate (**59**), ellagic acid (**60**), 1β,6-di-*O*-galloylglucose (**61**), mucic acid 1,4-lactone methyl ester 5-*O*-gallate (**62**), and mucic acid dimethyl ester 2-*O*-gallate (**63**) were isolated from the fruits of *P. emblica* L. [12,33]. The gallotannins corilagin (**64**) and geraniin (**65**) (Figure 3) were also isolated from *P. niruri* [34], and *P. muellerianus* [14], respectively.

### 2.4. Flavonoids

Compounds **66**–**69** (Figure 4) are kaempferols, which contain one kaempferol (**66**) and three kaempferol glycosides (**67**–**69**), and were identified from *P. acidus* [35]. Quercetin (**70**) and its glycoside rutin (**71**) were found in extracts of both *P. niruri* L. [10] and *P. amarus* [36]. Two new chalconoid analogues with anti-tobacco mosaic virus (TMV) activity, namely emblirol A (**72**) and B (**73**), and flavanol catechin (**74**) were isolated from the roots of the *P. emblica* L. [33].

### 2.5. Alkaloids

Five securinega alkaloids including (+)-allonorsecurinine (**75**), ent-norsecurinine (**76**), nirurine (**77**), bubbialine (**78**), and epibubbialine (**79**) (Figure 4) were isolated from *P. fraternus* G.L. Webster [37].

### 2.6. Other Compounds

Compound **80** (Figure 4), belonging to the naphthalene acetic acid class, is present in *P. amarus* and *P. urinaria* [24]. Moreover, constituent **81** (PEPW80-1) belongs to the polysaccharides, and was isolated from *P. niruri* [38]. HPLC–MS is a powerful analytical tool, which could efficiently characterize the chemical composition of various *Phyllanthus* species. Detailed and pioneering explorations of the phenolic compounds in *P. acuminatus* L. and *P. niruri* L. were done, which unravelled the presence of nearly 40 different phenolic derivatives for the first time in these *Phyllanthus* species, including pinocembrin isomers, apigenin derivatives, chrysin, quercetin, kaempferol, ellagitannins, prodelphinidin B dimer, *epi*-gallocatechin, ellagic acid, trimethylellagic acid, and ferulic acid [39,40]. One recent extensive study confirmed the presence of various phenol, flavonoid, non-flavonoid, tannin, alkaloid, saponin, and phytosterol phytochemicals in dried *P. emblica* fruits through the use of various extraction solvents (diethyl ether, ethyl acetate, butanol, and water). Furthermore, many low molecular weight aliphatic acids, phenolic acids, methyl/ethyl gallate, phytosterols, and tannins were identified in the fruit extracts [41]. A total of 51 compounds including phenolics, flavonoids and terpenoids were identified and tentatively characterized in ethanolic extracts of four *Phyllanthus* species (*P. amarus*, *P. niruri*, *P. emblica* and *P. fraternus*), while 23 compounds were also simultaneously quantified through UPLC-ESI-MS [42,43]. Recently thirty compounds, including derivatives of quercetin, kaempferol, epicatechin, coumaric, and cinnamic acids were identified in 50% ethanolic extracts of *P. acidus* leaves [44].

## 3. Biological Activities

### 3.1. Parasitology

The study of the parasites and their relationships with the host, called parasitology, has been a quite vast area of research in the last few decades. A hige number of studies have focused on plants to find active ingredients that can fight various pathogens and stop their activity in the host. *P. fraternus* G.L. Webster (Phyllanthaceae) is enriched with various alkaloids such as the securinega alkaloid (+)-allonorsecurinine and many other previously known alkaloids. These compounds showed highly antiplasmodial activity against chloroquine-resistant (W2) and -sensitive (3D7) strains of *Plasmodium falciparum* [37]. Higher oxidative stress features prominently in the pathogenesis of malaria, especially anaemia and patho-physiological modifications in certain body organs. Seed extracts of *P. amarus*, chloroquine (CLQ) and artesunate (ATS) may effectively reduce this oxidative stress alone or in combination with various vitamins (A, B, C and E) in *Plasmodium berghei*-infected mice. The highest antioxidant activity was shown by *Phyllanthus* seed extracts alone or in combination with vitamins (A, B, E) in *P. berghei* (NK 65 strain) infected mice. The combined activity of artesunate/vitamins also showed an enhanced antimalarial activity due to their antioxidant activity, while combination with vitamin C was counterproductive [45].

Human schistosomiasis is an important but neglected disease in tropics caused by blood flukes (*Schistosoma* spp.), which affects around 0.3 million people annually. The only known reported treatment is the use of paraziquantel (PZQ). De Oliviera and colleagues tried crude hexane (HE) and ethanolic (EE) extracts of *P. amarus* in mice infected with *Schistosoma mansoni* (BH strain) [6]. The in vivo schistosomicidal activity evaluation of mice fed with extracts of *P. amarus* once, for two different infection periods at 30 and 45 days post-infection shiwed that histopathologically, granuloma decreased in both number and size for groups treated with 250 mg/kg of HE (45 dpi) or EE (30 or 45 dpi). Both HE and EE of *P. amarus* have antischistosomal activities, however, they act differentially according to the parasites’ age. The schistosomicidal activity results in groups treated 30 days post-infection is extremely important since praziquantel does not show any activity against the juvenile forms of *Schistosoma* spp. [6]. *P. amarus* HE and EE extracts showed promising results against *S. mansoni* in vivo [6].

The dried fruits of *Emblica officinalis* (syn. *Phyllanthus emblica*) showed antitrypanosomal activity and cytotoxic effects in vitro. Vero cell line cells were incubated with *Trypanosoma evansi* over 12 h and treated with various concentrations (250~1000 μg/mL) of *E. officinalis* for an in-vitro cytotoxicity assay. A sharp decrease in trypanosome number was observed at 250 μg/mL concentration, and trypanosomes were completely killed after 5 h of treatment. This is statistically equivalent to the 4th hour of diminazine aceturate (Berenil) treatment, the standard reference drug used. *E. officinalis* dried fruits demonstrated a potential pathway for the development of new trypanocides if in-depth investigations were to be put in place [46].

### 3.2. Cardiovascular Protection

A large number across across the globe suffer from heart/stroke attacks, mainly due to poor life styles and increased sugar/carbohydrate intakes. In a recent study, the cardioprotective action of aqueous extracts of *P. amarus* was studied against high-sugar (fructose) diet-mediated cardiac damage in Wistar rats, Following 60-days of sugar diet, heart and aorta tissue samples were collected for further histopathological and biochemical analyses. Coadministration of *P. amarus* aqueous extracts plus glucose-diet for a specified time period (60 days) inhibited cardiac and aortic lipids levels (total lipids, triglycerides, total cholesterol and free fatty acids) and reduced phospholipid formation [36]. Histopathological evaluations of the heart and aorta tissues highlighted that the plant aqueous extracts treatment lessened the deposition of fats and necrosis. This study showed the obvious cardioprotective potential of *P. amarus* aqueous extract for treatment of high sugar-diet mediated oxidative stress in rats is mainly due to its ameliorative antioxidant potential along with its antihyperglycemia and antihyperlipidemic properties [36]. Moreover, the phytochemical profiling of the aqueous extracts of *P. amarus* indicated it may contain the flavonoids quercetin and rutin, the lignans phyllanthin and hypophyllanthin, saponins and the phenolic compound gallic acid, all of which are potent drivers of cardioprotective action [36]. In another study, a combined ethanolic extract of *P. emblica* fruits with an ethanolic extract of *Alpinia galanga* rhizomes (7:3) showed a strong synergistic antioxidant response against various reactive oxygen species in endothelial cells (ECV304) in a dose-dependent fashion, and caused an increase in cell viability. It was found that the potent and active ingredient is quercetin, which at 10 μg/mL concentration reduced the H_2_O_2_-mediated lipid peroxidation. When combined with ellagic acid and hydroxycinnamic derivatives, quercetin may aid antioxidant-mediated cytoprotection [47].

### 3.3. Antioxidant Activity

Antioxidants are active compounds that have the potential to mitigate the oxidation and degradation of various cellular proteins and ingredients by reactive oxygen species (ROS). Antioxidants play a crucial role in good health as in at-rest conditions ROS and antioxidants maintain a steady balanced level in the body [8]. Medicinally important flora have played a role in resolving health issues throughout the world for centuries, and have currently gained international focus in recent decades. The presence of antioxidant molecules in plants is well documented and there is an ever increasing demand for natural antioxidants over synthetic additives [48]. Normal metabolism of oxygen and exogenous factors continuously generates free radicals i.e., reactive oxygen species (ROS) which may initiate a cascade of chemical reactions that could be damaging to the human body [49]. Once these chemical cascades are initiated in vitro they may produce damage that leads to a decrease in number of viable cells [50]. The fruit and leaves extract of *P. acidus* have been reported to possess a variety of antioxidants, which are well known to quench ROS generated by the cellular metabolism, and check oxidative stress-mediated ailments such as cardiovascular diseases and inflammation [51]. Moreover, leaf water extracts of *P. amarus* are desirable due to their maximum yield of the biologically potent antioxidant compounds for in vitro antioxidant activity, ROS scavenging and inhibition of lipid peroxidation [52]. Singh and colleagues studied ethanolic and aqueous extracts of *P. niruri* to find antioxidant activity by using DPPH (2,2-diphenyl-1-picrylhydrazyl) radical and H_2_O_2_ scavenging assays [50]. A standard antioxidant (ascorbic acid) was used as control to compare the aqueous extract antioxidant efficacy and confirmed the strong antioxidant activity, however, ethanolic extracts of *P. amarus* and *P. niruri* showed higher ROS scavenging response compared to aqueous extracts [9,50,53]. Many studies have confirmed the presence in *P. niruri* extracts of quercetin, which is considered as potential natural antioxidant which is even more powerful antioxidant than vitamin E [49,54]. *P. acidus* fruit ethanol and water extracts were also studied to find antioxidant and cytotoxic levels, which showed that water extracts are more potent antioxidant and cytotoxic compared to fruit ethanolic extracts [55].

Different solvents *viz*. acetonitrile, alcohols like ethanol and methanol, water, ethyl acetate, and dichloromethane were used through multiple extraction methods such as conventional, ultrasound-assisted, microwave-assisted, etc. to determine the extraction efficiency and antioxidant capacity of *P. amarus* [11]. Moreover, water and microwave-assisted extraction has been demonstrated as the most efficient solvent and technique for the maximum isolation of biologically active compounds from *P. amarus* for wide application in industry [11]. The aqueous extracts of three endemic Cuban *Phyllanthus* species, *viz*. *P. chamaecristoides* Urb., *P. microdictyus* Urb., and *P. williamioides* Gr. were examined to determine their antioxidant, photoprotective and antimutagenic activities [16]. This study claimed that DNA damage was reduced by *Phyllanthus* aqueous extracts and is not linked with desmutagenic effect in vitro, and genoprotective activity is due to the induction of expression of DNA repair proteins, reduction of ROS and related mechanisms [16]. Among the various *Phyllanthus* species, *P. chamaecristoides* extracts have higher antioxidant behaviour than those of *P. microdictyus* and *P. williamioides*. It is generally believed that the higher antioxidant potential of any compound ultimately lowers the UVR mediated oxidative damage [16].

Zhang and co-workers [56] reported two new compounds (bisabolane-type sesquiterpenoid diphenyl ether derivatives) along with previously 23 known compounds from the fruit extracts of *P. emblica*. These two new isolates were tested for antioxidant capability through DPPH assays and for cytoprotective effects and showed activity against H_2_O_2_-mediated injury to PC12 cells [56]. 

Long term exposure to pesticides may cause severe effects to most body organs, which have been attributed to an elevation of ROS and genomic DNA damage. *P. emblica* was also reported to be beneficial for in vivo protection against the effects of fungicidal pesticides such as captan, that causes genotoxicity and help generate ROS [18]. The extracts could mitigate captan-mediated oxidative stress and genotoxicity which is presumed to be due to the potential antioxidant activity of the *P. emblica* extracts. *P. indofischeri* leaves and bark extracts were prepared in water and ethanol, and found to possess significant α-amylase inhibitory and antioxidant activity [57]. The methanol leaf extracts had better activity than bark extracts, hence providing a clue that *P. indofischeri* can be a a useful addition to use as a potent antioxidant and hypoglycemic candidate to combat ROS-mediated ailments. The methanolic leaf extracts of *P. emblica* were examined, and it was said that the polyphenolics present in it may provide strong protection against lipid peroxidation damages, and increased levels of SOD, glutathione peroxidise and catalyse enzymes are primarily due to the presence of gallic acid, rutin, caffeic acid, and kaempferol [58,59].

*P. muellerianus* (Kuntze) Exell., is used as a wound healing agent in different African countries. Geraniin, a powerful antioxidant, was identified as a major constituent in the aqueous extracts of *P. muellerianus* that help reduce oxidative stress and boost the healing process in chronic wounds [60]. The effects of geraniin in treating chronic wounds are mainly due to elevation of SOD, CAT and APx levels, and decreased malondialdehyde (MDA) levels at the wound site. Moreover, geraniin significantly and efficiently reduced ferric ion in vitro, which helps to blockage of the iron-mediated amplification of ROS [60]. The ethanolic leaf extracts of *P. amarus* were studied to find the cause of modification in serum antioxidant levels along with ROS-mediated MDA in mice, and it was reported that the antioxidant defense capacity and revitalization of the blood in treated mice was increased, which might due to the presence of previously reported compounds [48]. *P. urinaria* has a well known history in traditional medicine systems to treat cancers, particularly osteosarcoma, which is one of the most aggresive cancers of bones. Osteosarcomas originally arise from the primitive transformed cells of mesenchymal origin that form malignant osteoids [61]. The aqueous preparations of *P. urinaria* affect human osteosarcoma in vivo and significantly reduce the tumorigenesis, but have no effect on the rest of the body organs. The reduction in tumor development was suggested to mainly be due to mitochondrial dysfunction linked with dynamic changes brought up by apoptosis and anti-angiogenesis induced by *P. urinaria* [61]. The in vitro anti-oxidant and anti-proliferation potential of the water extracts made from the aerial parts and roots of *P. debilis* be studied with special focus on the role of polyphenolics and its broad utilization. The roots of *P. debilis* have more flavonoid contents than the aerial parts [62].

### 3.4. Anticancer Activity

Cancer is a condition where the certain types of cells mutate and start proliferating unevenly. Almost every kind of human cancer evolves due to a loss of normal cellular physiology. Moreover, cancer is always a big challenge to cure, and avoiding its recurrence following treatment, whether by using chemotherapy, phototherapy, or combinatorial therapies, needs more research for the exploration of potential anticancer drugs from natural sources. The plants in the genus *Phyllanthus* are well-known to possess medicinally active ingredients and have long been used as traditional antitumor remedies throughout the world [63]. Various chronic diseases such as cancer, hepatitis and diabetes mellitus have been well treated with *P. amarus* extracts in traditional medication systems in China [52]. It was further explained that *P. amarus* extracts have potential to be utilized at a commercial scale for the isolation of natural antioxidants and some novel anticancer agents [52]. *P. amarus* extracts have good anti-tumor activity and good drug-herb interactions with 5-fluorouracil (5-FU), and at higher concentrations show greater toxicity to HepG2 cells by inducing G2/M cell cycle arrest or modulation of major enzymes involved in intracellular ribonucleotide and deoxyribonucleotide metabolism [64]. Apoptotic cells are one of the main biomarkers used to predict and define what type of treatment should be given to patients suffering colorectal cancer and monitor the end results. *P. niruri* L. acts as an antineoplastic agent, and its extracts increase the apoptosis of colorectal cancer in vivo and in vitro [65]. Moreover, the antitumor components of *P. niruri* have been reported for the first time and the presence of ethyl brevifolincarboxylate and corilagin identified. *P. niruri*-derived corilagin has wide antitumor potential on HCC cells and the results proved it is a superior antitumor agent causing the least harm to normal and neighbouring cells [63]. 

*P. emblica* L. is traditionally being used to treat various ailments in the Southeast Asia since centuries gone. It is believed to prevent tumor formation linked with nonresolving inflammation possibly due to anti-inflammatory effects of *P. emblica* extracts against precancerous lung lesions. Exposure to *P. emblica* extracts has significant reduction in nodal counts on lung surface and diminished B(a)P-induced expression of proinflammatory cytokines such as MIP-2, TNF-α, IL-6, and IL-1β in lung tissues along with protein expression of HIF-α and COX-2 [66]. Moreover, it was stated that treatment with *P. emblica* extracts provides strong protection to the lung tissues from inflammation linked injuries but also efficiently check precancerous lung lesion formation by modulation of IL-1β/miR-i101/Lin28B signalling cascade [66]. *P. emblica* extracts may induce the activation of the nuclear related factor 2 (Nrf2) oxidative stress signal pathway through the involvement of Extracellular signal-Regulated Kinase (ERK) and p38 mitogen-Activated Protein Kinase (p38MAPK) in HepG2 cells. Nrf2 pathway activating mechanisms of *P. emblica* extracts might be linked with the roles of ERK and p38MAPK via phosphorylation and nuclear translocation and accumulation of Nrf2 [67]. The cytotoxic effects of various leaf extracts (aqueous, methanol, ethyl acetate, petroleum ether, chloroform:methanol and water: methanol) of *P. emblica* L. were studied, and it was determined they are primarily due to the presence of two well known major flavonoids i.e., kaempferol and rutin, which are used as chemotherapeutic agents [68]. *P. emblica* extracts plays a role in delaying mitotic progression along with enhanced genomic instability (GIN) in human colorectal cancer cells. The plant extracts were applied to examine the effects on spindle assembly checkpoints, mitotic aberrations and GIN in human NCM460 normal colon epithelial cells. It was revealed that plant extracts provide protection to human normal colon epithelial cells from mitotic and genomic damages partly by increasing the role of spindle assembly checkpoints [17].

Mitomycin C and cisplatin have been extensively used as chemotherapeutic drugs but certain limitations (genotoxicity) for normal cells limit their use. *P. emblica* extracts reduce this genotoxicity, improving the anticancer effects of both these drugs along with inhibition of clonal expansion of unstable genomes in normal cells [19]. *P. emblica* is claimed to reduce the danger of onset of secondary cancers potentiated by various chemotherapeutics. The novel alkaloids securinine and allosecurinine have been reported to occur in *P. glaucus* (leaf and flower), and these possess anti-proliferation potential for various cancer cells. Securinine drives apoptotic pathways by activating cell cycle arrest at multiple points in HeLa cells, which is linked with ROS stress and the mitochondrial programmed cell death pathway [69].

### 3.5. Anti-Aging and Skin Protection Properties

Sunlight ultraviolet (UV) radiation constitutes a significant physical carcinogen in Nature. Natural products or phytochemicals are being used for protecting human skin tissue against the harmful effects of solar ultraviolet (UV) radiation as photoprotectors. UVR could induce direct and indirect DNA damage, which if not properly repaired may generate mutations leading to abnormalities or tumorigenesis [16]. The extracts of *P. orbicularis* K. were examined as a photo-protective agent against photodamage caused by UVA and UVB through artificial lamp and natural sunlight exposures. The aqueous extracts have good UVR absorbing capabilities and hence can be used as photo-protective agents for GIN and DNA damages caused by environmental sunlight radiation [70]. Moreover, the use of *P. orbicularis* extracts reduced the CPD formation and oxidative damage in UV-irradiated DNA samples. The photoprotective effect of *Phyllanthus* extracts was more obvious for UVB damages compared to damages caused by UVA [70]. Moreover, recent investigations support the idea of a genoprotective activity of *Phyllanthus* species against chemical and physical mutagens, among them UV radiation [16]. Altogether, these studies will help define future pre-clinic research about the photoprotective properties of *Phyllanthus.* The in vitro anti-aging properties of *P. emblica* L., *Manilkara sapota* L. and combinations of both extracts were investigated and showed that they may have well defined in vitro antioxidant, anti-collagenase and anti-elastase activities useful for the cosmetic industry [71]. The extracts combination had no additional effects, but rather synergistic effects appear due to the higher antioxidant, anti-elastase and anti-collagenase potential of these extracts hence making them appealing as a sustainable source for the cosmetic industry in the future [71].

### 3.6. Antidiabetic Activity

Traditional or herbal medication systems are well proven to effectively control with potential therapeutic characteristics moderate declines in hypoglycemia, to cure and lower obesity as well as diabetes. Globally, a large population suffers from diabetes mellitus (DM), and their number is increasing annually along with diabetes-linked complications. Diabetes mellitus is a dysregulation in food (proteins, carbohydrates and fats) metabolism by insulin production in beta cells. DM is considered as a chronic metabolic dysregulation with a strong socioeconomic impact throughout the world. Many active constituents such as quercetin, gallic acid, and gallotannins present in *Phyllanthus* plant extracts were reported to interact with DM-associated protein targets, e.g., glycogen phosphorylase and peroxisome proliferator-activated receptor gamma (PPARγ). Quercetin being the major constituent in methanolic extracts of *P. emblica* fruits, has good binding ability to ligands and DM-linked protein targets, hence showing a potential anti-diabetic and antihyperglycemic activity through modulation of cholesterol, glucose, and triglycerides [72]. Root extracts of *P. watsonii* A. Shaw were prepared in three different solvents (petroleum ether, chloroform and ethanol), and showed strong and dose dependent antidiabetic effects and in vivo antioxidative activities in type 2 diabetic rats [73]. The aqueous extracts of *P. niruri* were thoroughly studied for their physicochemical characteristics and organoleptic characters, and it was described that the displayed high antidiabetic activity is mainly due to the presence of phenolics in the extracts [74]. *P. emblica* showed a significant reduction of glucose levels in blood plasma within 30 days of administration to streptozocin fed diabetic rats [75]. The fruits, leaves and flowers of *P. emblica* are rich in ellagitannins have been stated useful to ameliorate diabetes when tested in streptozotocin fed rats. *P. emblica* aerial part extract consumption aids in the vascular function in hyperglycemic rats by regulating Akt/β-catenin signaling, and the effects are potentially mediated by the ellagitannin metabolite urolithin A [76]. The known chemical treatment methods for diabetes also have some side effects. It is suggested that *P. fraternus* methanolic leaf extracts contain active ingredients that are able to reduce glucose levels in streptozotocin fed rats [77].

Traditional medicine systems have mentioned the excessive use of *P. niruri* to cure severe diabetes. The ethanolic and aqueous extracts of aerial parts of *P. niruri* showed α-glucosidase inhibitory activity, primarily due to the presence of corilagin and repandusinic acid A [78]. The ethanolic extracts of *P. niruri* has no any actions to lower glucose-6-phosphatase activity, rather it raised deoxyglucose up-take in C2C12 muscle cells and enhanced adipogenesis in 3T3-L1 fat cells which is a pioneering finding that could be useful to treat type 2 diabetes mellitus [78]. In another study, *P. niruri* has been stated to lower a particular type-2 DM and obesity by decreasing sugar levels in blood serum while maintaing a healthy lipid profile status in obese diabetic rats [79]. The seeds were removed from *P. emblica* fruits to prepare aqueous and methanolic extracts, which surely reduced the blood glucose levels in oral glucose tolerance tests in the mice. It is further advised that *P. emblica* fruit extract in combination with *Trigonella foenum-graecum* seed extract could be a very desirable and beneficial alternative medicine for reducing blood glucose levels in hyperglycaemic patients or those with severe diabetes [80]. Chronic oxidative stress caused by ROS is thought to be the major cause of diabetes. ROS-mediated oxidative stress may also hurt pancreatic cells, and *P. amarus* extracts are claimed to provide cytoprotection to pancreatic cells in vivo in streptozotocin-induced diabetic rats, mainly by reducing this ROS mediated oxidative stress [81]. Moreover, *P. amarus* plant extracts could potentially be used as an additive for managing and treating diabetes [81]. *P. acidus* leaf extracts have also strong hypoglycemic effects on normal rats and orally glucose-induced hyperglycemic rats compared with distilled water and glibenclamide [82].

### 3.7. Organ Protective Effects

Among many other ailments, renal disease is considered as the 9th major cause of mortality across the world, and is considered the sole clinical sign of this disease. The disease is characterized by reduced excretory function by the kidney, and hence reduced glomerular filtration rate (GFR), with abnormal homeostasis in blood chemistry. Renal failure disease is mainly caused by environmental pollution, particularly by heavy metals which pose a treat to the biosphere and and particular to people exposed to industrial wastes or effluents, or farm workers, primarily attacking the kidneys. For instance, cadmium (Cd) enters the kidneys where it deposits in the proximal tubules [83]. Cd causes a decline in kidney activity by elevating blood urea and creatinine levels, which leads to further kidney damage by exposure to leaf extracts of *P. amarus*. It was suggested by Olubunmi and colleagues [83] that *P. amarus* extracts have no prophylactic or ameliorative effects on Cd-mediated kidney damage, and rather that continuous exposure to these extracts are deleterious to the kidney, but the Ayurvedic system of medication reports that *P. emblica* fruits are potent herbs, and hydroalcoholic extracts provide protection against hepatic disorders. Ethanolic extracts of fruits of *P. emblica* provide hepatoprotection mainly because of the high ROS scavenging and antioxidant activity of their main constituents like gallic acid and ellagic acid [84,85]. In different human diseases like diabetes, obesity and cardiovascular issues, insulin resistance is increased which is primarily due to higher intake of sugars. The ethanolic extracts of *P. amarus* are quiet beneficial and ameliorate insulin resistance by reducing metabolic syndrome and hepatic ROS levels in rats [36]. Ethanolic extracts of *P. emblica* L. dried fruits, have been reported to show positive gastroprotective effects in patients by reducing pain, vomiting, insomnia and sleeping disturbancess or associated problems [86]. Water extract of *P. emblica* potentially reduced lipid peroxidation, mRNA expressions of CYP2E1, TNFα and IL-1β, while increasing antioxidant activity and reducing non-alcoholic steatohepatitis in vivo [13]. However, although *P. niruri* extracts are enriched with anti-oxidant and anti-inflammatory activities, no any significant clinical benefits were observed in non-alcoholic steatohepatitis treatment [87]. The fruits extracts of *P. emblica* have ellagic acid as the major component, which helps reduce the ROS generation and fat accumulation, while modulating the expression of lipogenesis- linked genes and up-regulating AMPK signaling in HepG2 cells. Extracts showed an inhibition in cellular steatosis and liver fibrosis in vitro [88].

### 3.8. Diuretic Effects

Diuretic medicines are of great importance in treating many cardiovascular and renal diseases including hypertension, heart failure, eclampsia, nephritis and chronic renal failure. Drugs with diuretic effects may aid in the regularization of blood pressure, facilitate urinary excretion, decrease blood volume, remove protein metabolites and toxic compounds. The excessive use of diuretics is not recommended due to certain limitations caused by their side effects, particularly electrolyte imbalances, metabolic alterations, activation of the renin-angiotensin system (RAS) and neuroendocrine and sexual function disturbances, all of which guide the search for novel diuretics from natural sources without any such drawbacks. *P. amarus* aqueous extracts have strong effects on urinary excretion. The aqueous exctracts of *P. amarus* were further partitioned into ethanol and chloroform extracts, of which the ethanolic fraction (furosemide) had more diuretic potential dieresis [89]. Furthermore, the ethanolic fractions of *P. amarus* are also said to promote urinary excretion of water and Na^+^, thus confirming its diuretic activity which is linked with prostaglandins [90].

### 3.9. Brain Functions

The brain is the control tower of all the body functions, and implements all of its orders through the neurons or sensory cells to perform specific functions. Proper brain function is necessary for a healthy life. Many food ingredients and certain phytochemicals that boost the brain function are well known and are well studied. With the passage of time, when a person is getting older, most of the nerve cells undergo progressive and gradual degradation that results in neurodegenerative diseases, hence impairing the normal and healthy functioning of the body. A very familiar example of such nerve cell damage is Alzheimer’s disease, which results from oxidative damage caused within nerve cells, and can be ameliorated by using natural antioxidants to slow the pathological progression. Remarkable and beneficial effects have been reported in a study where *P. emblica* fruit extracts improved not only the antioxidant potential, but also the learning and memory in rats [91]. Moreover, *P. emblica* fruit extracts could help various cognitive disorders, dementia, and neurodegenerative disorders. *P. acidus* is said to help reduce inflammatory pains and oxidative stress-related disorders, and provide neuroprotection to nerve cells. Methanolic extracts of *P. acidus* reduce oxidative stress and enhance cognitive function by increasing brain antioxidant enzymes, decreasing lipid peroxidation and displaying anti-acetylcholinesterase activity, being used to treat oxidative stress-mediated Alzheimer’s disease (AD). *P. acidus* fruit extracts can be a potent and novel therapeutic approach to treat neurodegenerative dementia, especially AD [92].

*P. emblica* has positive effects on retinal degeneration in a well-defined animal model of AD. *P. emblica* extract inhibits intracellular ROS to reduce the oxidative stress and severity of histological changes caused by AD-induced retinal degeneration in retinal tissue. Moreover, the main plant extract ingredients include phenolic compounds such as hydrolysable tannins and their glycoside derivatives, that actively regulate neurofilament (NF)-L, thymocyte differentiation antigen 1 (Thy-1), and sirtuin 1 (SIRT1) levels in the retinal tissue [93]. It is further said that ellagic acid activates intracellular antioxidant enzyme activity and raises cell survival in astrocytes [93].

### 3.10. Analgesic and Anti-Inflammatory Activity

Inflammations are the immune responses to any disturbance in cells or tissues under stress. Prolonged exposure to these various stresses such as pathogens, chemicals, UVR, and pollutants, etc. may lead to serious inflammation that leads to chronic diseases. Inflammation and pain are quiet frequent causes of medical discussion which generally occurs when a tissue is injured [94]. Recently much focus has been devoted to screening and isolating novel drugs with analgesic potential from various plant sources in order to minimize pain or inflammation with less side effects compared to Western medicines [94]. The members of the genus *Phyllanthus* are well studied for their analgesic and anti-inflammatory effects. In a recent study where Swiss albino mice were evaluated for the anti-inflammatory and analgesic properties of leaf ethanolic extracts of *P. acidus*, and it was observed that these extracts displayed remarkable action against inflammation and pains when applied at a final concentration of about 200 mg/kg of total body weight [95]. A comparative study to find anti-inflammatory, anti-arthritic and analgesic activity of various herbal extracts of *Bacopa monnieriis*, *Cassia fistula* and *P. polyphyllus* was conducted by Yoon and Lee [96], which claimed that the extracts in various combination (*w*/*w*/*w* = 1/2/1) had significant anti-inflammatory and analgesic actions.

The aqueous extracts of the aerial parts of *P. muellerianus* along with its prominent secondary metabolites, e.g., geraniin, were found to have potential both peripheral and central anti-nociceptive effects in murine models of chemical nociception with the anti-nociceptive action of geraniin involving possibly the opioidergic pathways [14]. The green synthesis of silver nanoparticles (AgNPs) using aqueous fruit extracts of *P. acidus* represents an environmentally friendly and cheaper source of a materials with potential therapeutic roles in cytoprotection and anti-inflammation through scavenging nitric oxide and superoxide anions [97]. Furthermore, short-term exposure to *P. acidus*-mediated green-synthesized AgNPs did not affect the viability of peritoneal macrophages, and could be a potential therapeutic to treat inflammatory diseases by reducing the expression of IL-1β [97].

The central nervous system (CNS) depressant activity was measured along with antidiarrheal and antipyretic activities of ethanolic leaf extract of *P. acidus* L., which reduced CNS depression significantly in animal models in a dose dependent way [98]. Moreover, *P. acidus* showed significant antidiarrheal and antipyretic actions, and therefore it could be an excellent source for natural CNS depressant, antidiarrheal and antipyretic agents for medical applications [98]. The comparative therapeutic efficacy of *P. emblica* fruits extract and procaine penicillin in the treatment of subclinical mastitis was studied in 30 subclinical mastitis positive buffaloes. It is concluded that *P. emblica* fruit extract is an inexpensive source to treat subclinical mastitis in dairy buffaloes and can be used as an alternative to antibiotic therapy like procaine penicillin [99]. In inflammatory ulceration *P. niruri* is commonly applied in traditional medication systems to treat ulcers and inflammation. The methanolic extract of *P. niruri* leaves was used for its anti-inflammatiory and anti-ulcer activities, which suggests that leaf extracts are strong enough to reduce inflammation and provide protection against ulceration, as ascertained by regeneration of mucosal layer and substantially prevented hemorrhage and edema [15].

Another study recently evaluated the anti-nociceptive and anti-inflammatory potential of phytosterols isolated from the chloroform extracts of *P. maderaspatensis* (CEPM) through the carrageenan-induced hind paw oedema and hot plate method in male Wistar rats. CEPM extract and pentazocin had significant effects on the increase of the basal reaction time compared to control. This demonstrated the potential anti-inflammatory and analgesic effect of the CEPM which supports the claims by traditional medicine practitioners [94]. Significant hypoglycemic, anti-diarrheal, analgesic, and anesthetic activities were shown by *P. acidus* pulp extracts [100].

### 3.11. Immunomodulatory Effects

The human immune system is an organized system comprised of many different immune cells such as macrophages, neutrophils, T-lymphocytes, natural killer cells and various other specialized immune molecules that have evolved to mediate resistance against infections. Wide ranging ethnomedicinal or traditional uses of *Phyllanthus* plants are mainly linked to its active ingredients and broad pharmacological actions, e.g., immunomodulation, anti-viral and antibacterial, diuretic, anti-hyperglycemia and hepatoprotector properties [101]. Phytochemicals used as immune- stimulants, are superior to conventional chemotherapeutics and antibiotics. Tuberculosis (TB) is a severe disease, and its progression mainly depends on the strength of the host immune system. *P. niruri* has been reported to boost the immune system in traditional medicine, and it is said that it has a great potential to induce immune cell activity in TB patients in vitro, The *P. niruri* extracts mainly help the release of nitric oxide and hence elevate the phagocytic activity of macrophages in a dose dependent fashion, ultimately modulating the immune responses [102]. Moreover, *P. niruri* leaf extracts have immunostimulating effects on neutrophil activation and the antibody response of *Oreochromis mossambicus*. It is further demonstrated that *P. niruri* plant extracts and their components can be used either as a routine feed supplement to activate the immune system of farmed fishes or as an adjuvant to enhance the efficacy of vaccines [103]. *P. amarus* strongly inhibits the phagocytic activity of human neutrophils and reduces cellular immune responses in rats. Moreover, the plant extracts have strong inhibitory effects on cellular and humoral immune responses (ceruloplasmin and lysozyme) suggesting the potential of the plant to be developed as an effective immunosuppressive agent which mainly acts through the inhibition of myeloperoxidase activity and nitric oxide release leading to the release of serum level immunoglobulins [104]. Phyllanthin, one of the main and active ingredient in many *Phyllanthus* species (*P. amarus*), has recently been reported to have hepatoprotective and immunosuppressive effects on various in Balb/C mice in a dose-dependent way through the inhibition of CD11b/CD18 adhesion, nitric oxide and myeloperoxidase activity release [105]. Based on the level of doses of phyllanthin, a significant inhibition in the proliferation of B and T lymphocytes and down-regulation of Th1 (IL-2 and IFN-γ) and Th2 (IL-4) cytokines, CD4+ and CD8+ was noted [105].

### 3.12. Antibacterial Activities

The ability of a diverse class of microbes to resist against known anti-microbial drugs is always been a universal problem leading to explore novel anti-microbial drugs from diverse natural sources such as medicinal plants, expecially the medicinal flora which have not been examined for their pharmacological potential as anti-bacterial potentials.

*Phyllanthus niruri* L. is one of the plants that can be used as a preventive and alternative treatment as a substitute of antibiotics for the treatment of Chronic Respiratory Disease (CRD) in broiler chickens caused by *Mycoplasma galisepticum*. The chemicals contained in meniran (*P. Niruri* L.) include antibacterial tannins, saponins, and alkaloids. A 30% plant extract caused up to 65% growth inhibition in *Mycoplasma galisepticum* [106]. In continuation of a similar study, Ramandeep and colleagues found that *P. niruri* extracts could potentially inhibit the growth of *Escherichia coli* (*E. coli*), *Lactobacillus acidophilus* (*L. acidophilus*), *Pseudomonas aeruginosa* (*P. aeruginosa*) and *Staphylococcus aureus* (*S. aureus*). Moreover, *P. niruri* is rich in phytochemicals that have antioxidant and antimicrobial activity, suggesting the need for further in-depth investigations for its systematic use in traditional medicine [21].

The extracts from *P. amarus* leaves were examined for a complete chemical analyses along with their anti-microbial potential. Various stable extracts, i.e., hexane (59%), acetone (57%), and water extracts (48%) were prepared, which contain many alkaloids, saponins, anthraquinones, tannins and phenolics in acidic medium. In general, combining leaf extracts with either bacitracin or erythromycin alone has synergistic effects, thus depicting the crucial advantage of such combinations of these universal antibiotics with extracts of *P. amarus* to treat various infections [107]. Various other studies examined the anti-microbial activities for aqueous extracts of above ground parts of *P. muellerianus*, and it was stated that geraniin is the major component in these extracts. In certain an agar-well diffusion assay, micro-dilution method, and time-kill kinetic studies were applied to find the anti-microbial potential of areal plant parts extracts, where geraniin have strong inhibitory effects on *E. coli*, *P. aeruginosa*, *S. aureus*, *B. subtilis*, and two clinical isolates viz. *S. pyogenes* and *C. albicans*, and significantly inhibited the zones of growth. Moreover, the minimal inhibitory concengtrations of these aerial plant parts extracts and and geraniin were in a variable range i.e., 0.31 to 5 and 0.08 to 1.25 mg/mL, respectively. In another experiment where minimum cidal concentration were recorded for the plant extracts (5.0 to 50.0) and geraniin (2.5 to 10 mg/mL), respectively, thus proven that both the extracts and geraniin have strong anti-microbial potential. All these initial studies regarding indepth exploration of various chemical constituents in *P. muellerianus* extracts further confirmed the occurrence of various terpenes, flavones, saponins, tannins, alkaloids and glycosides. It is further said that, these extracts have geraniin as primer constituent which pose strong anti-microbial potential against the above mentioned bacterial strains [108].

In continuation of similar finding, a study examined the antimicrobial potential of volatile oils extracted from leaves of *P. muellerianus*, where they found good inhibitory effects partly on the isolated pure extracellular protease of pathogenic *Klebsiella granulomatis*. These volatile oils showed strong antimicrobial potential as depicted by their inhibitory capability against these proteases. Further the authors proposed that in-depth studies and clinical trials using these oils could define their effectiveness to combat various skin conditions such as third degree burns, cuts, injuries and post-operative wounds infections caused by *K. granulomatis* as well as many related strains [109].

Pathmavathi and Thamizhiniyan prepared various leaf extracts (methanolic, hexane, chloroform, and ethyl acetate) of *P. amarus* and *Plectranthus ambionicus* to study their antifungal and anti-microbial potential [110]. Four Gram positive and three Gram negative bacterial strains along with three species of fungi were tested to screen the best isolate among the various organic plant extracts. The anti-microbial activity of various extracts of both these plant species showed mixed levels of anti-microbial potential towards the selected fungal and bacterial strains, but the ethyl acetate extracts of *P. ambionicus* and *P. amarus* showed superior antimicrobial potential compared to other extracts. Recently zinc sulphide nanoparticles were synthesized from *P. emblica* leaves and fruit extract to analyse the activity of these significant phytochemicals. These zinc sulphide nanoparticles possess potential antimicrobial activity against many pathogenic organisms [111]. The anti-*Helicobacter pylori* and urease inhibitory activity of hydroalcoholic extracts of *P. niruri* L. was studied, and it was found that quercetin is one of the major constituents which is thought to be the cause of noncompetitive urease inhibition [10].

The leaf extracts of *P. amarus* showed inhibitory effects on *Streptococcus pyrogenes* (*S. pyrogenes*), *Streptococcus pneumonae* (*S. pneumonae*), *S. aureus*, *P. aeruginosa* and *Candida albicans* (*C. albicans*) in a study by Oluboyo and colleagues [112]. Many of the phytochemicals (flavones, terpenoides, various alkaloids, benzenoids, steroids, saponins and complex lipid molecules) were compared with ampicillin, gentamicin and pefloxacin, and observed to possess significant inhibition activity against the growth of selected studied microorganisms in a dose dependent fashion. Moreover, well known antibiotics had no significant inhibitory effect on the tested microorganisms compared to the leaf extracts of *P. amarus* [112].

Gunawan and colleagues [113] have reported that *P. niruri* extracts mainly contain alkaloids, triterpenoids and flavones, which are highly efficcient to treat dysentery, rheumatism, inflammation and gut worms in children in traditional medication systems. Furthermore, active triterpenoids in the chloroform extracts from the bark of *P. niruri* possess strong antibacterial activity and control the proliferation of *S. aureus* at an optimum concentration (1000 ppm) evidenced by a great 12 mm inhibition zone [113]. In another study, remarkable anti-bacterial potential against various fungal and bacterial strains was seen by using silver nanoparticles (AgNPs) obtained from a supercritical CO_2_ extract of *P. niruri* [114]. The phytochemical analyses of *P. amarus* leaf extracts were studied and presence of alkaloids, saponins, tannins, flavonoids, cyanogenic glycosides and steroids for antimicrobial activity.

The phytochemical examination of the leaf extract of *P. amarus* shows the occurrance of a variety of bioactive components: alkaloids, saponins, tannins flavonoids, cyanogenic glycosides and steroids in ethanol and ethyl acetate. The extracts of both these solvents were applied to find their potential as anti-microbial agents against *S. aureus*, *E. coli* and *C. albicans*, and were found pretty suitable for the production of advanced anti-microbial drugs [115]. The leaf methanolic extracts of *P. niruri* showed a well defined antimicrobial activity against *Coney lunata* and *Salmonella typhi*, and it was suggested by the authors that this plant is of great clinical importance and may potentially be used in the pharmaceutical industry [116]. Chewing gums were prepared having constituents of fruits extract of *P. emblica* and studied in detail for their potential in alleviating and soothing oral health problems [117]. Fruit extracts of *P. emblica* have great pharmacological potential against bacterial growth, inflammation, cellular oxidative stress induced by ROS, and various types of cancers as well as diffent oral diseases. The focus of the study was changes in the oral microflora in a gum-base-controlled crossover manner caused by the effect of *P. emblica* leaf extracts. Moreover, chewing gums prepared with the extracts of *Phyllanthus* fruits help to stimulate saliva production, producing a significant reduction in the recurrence of clinical oral microflora for a short time period, and are thus proven as a safer way of improving oral health [117].

### 3.13. Antiviral Activity

The in vitro anti-hepatitis B viral activity of *P. niruri* L. in HepG2/C3A and SK-HEP-1 cells was studied by Li et al. [118]. The ethanol fractions were analyzed and reported to be enriched with ellagic acid fractions which successfully inhibited the growth of HBV-infected HepG2/C3A cells compared to the isolated active compound that showed a half-maximal inhibitory concentration (IC_50_) of 120 μg/mL and had no effect on HBV DNA replication at the concentrations evaluated, hence failing to inhibit the reproduction of HBV [118]. *P. amarus* has been traditionally used for treating a variety of diseases including hepatic disorders due to the presence of anti-hepatic viral compounds such as phyllanthin and hypophyllanthin in different types of in vitro cultures of *P. amarus* [119].

Hepatitis B virus claims around a million human lives annually. Sarma and colleagues [120] attempted to explore a potent and efficient antiviral from *Phyllanthus* with a minimal risk of resistance for hepatitis B virus. Moreover, in this attempt the *Phyllanthus* active principles from among 93 phytochemicals were isolated to check the mechanism of action against hepatitis B virus reverse transcriptase (HBV RT), which is an active target for drugs used against HBV infections [120]. Furthermore, a comparative binding energy study proposed that lupeol acetate, a triterpene present in various *Phyllanthus* species (*P. reticulates*, *P. urinaria* and *P. niruri*), has higher binding energies for native (−7.95 kcal/mol) and M204V mutated HBV RT (−6.16 kcal/mol) compared with lamivudine used as control drug. Subsequently, lupeol acetate was screened for in silico ADME/tox properties, and was shown to possess better bioavailability with no toxic effects and could therefore be treated as a potential drug [120].

Paramyxovirus is a cause of Newcastle disease in birds. In an attempt to cure this disease, an in-ovo assay agaist this virus was performed using the leaf extracts (aqueous, methanol and *n*-hexane) of *P. amarus*. Further analyses showed the occurrence of various phytochemicals (phenols, alkaloids, tannins, flavones, steroids, saponins, and glycosides) along with antioxidants in the leaf extracts prepared in various organic solvents. Leaf extract concentrations of about 50 mg/mL were toxic to in-ovo assay embryos, and hexane extracts reduced the viral titre in a manner directly proportional to the increased concentration of plant extracts. It is further described that leaf extracts of *P. amarus* potentially reduced the growth of NDV virus *in*-*ovo* [121].

White spot disease is said to be the most frequent and deadliest disease in shrimps, being caused by white spot syndrome virus (WSSV). The attachment of the virus onto the cell surface is supported by various envelope structural proteins, which are a characteristic required for the development of a good antiviral drug [122]. Molecular docking and simulation analyses were conducted to find the attachment potential of phytochemicals isolated from *P. amarus*, and it was stated that major envelope proteins *viz*. VP26, VP28, and VP110, and a nucleocapsid protein VP664 of WSSV are responsive to them. The docking result reveals that the volatile compounds 2*H*-1-benzopyran-6-ol, 1,4-benzenediamine (*N*,*N*′-diphenyl) exhibited the highest binding energy with the envelope proteins. Molecular dynamic and simulation studies showed the mobility behaviour of various protein–ligand binding complexes at different times. Therefore, *P. amarus* phytochemicals are said to be most suitable to inhibit and treat WSSV viral infections [122]. Another study where researchers applied *P. amarus* extracts to freshwater crab (*Paratelphusa hydrodomous* Herbst) by injecting them with three different solvent extracts (ether, acetone and aqueous) along with WSSV virus demonstrated the antiviral activity against WSSV. The authors claimed that *P. amarus* extracts prepared in ether and acetone have strong antiviral effects in WSSV viral infections [123].

Zhang et al. [124] investigated in vitro anti-HIV-1 activity and its related mechanisms of action of an extract isolated from *P. urinaria* to develop an HPLC methodology for detecting gallic acid (GA) in plasma and tissues to study its pharmacokinetics and tissue distribution in rats. The Biacore study indicated that polyphenols (PE) and GA present in *P. urinaria* interacted with HIV-1 RT, gp120, and P24. The PE isolated from *P. urinaria* containing GA has anti-HIV-1 activities. GA is quickly absorbed and slowly eliminated in rats after oral administration. The pharmacokinetics of GA administered as a PE is desirable, and it is widely distributed in the main tissues of lung and liver. Both its properties and anti-HIV-1 activities make it of interest for further studies [124]. Recently two new chalconoid analogues, i.e., emblirol A (**72**) and emblirol B (**73**), along with three previously known compounds (emblirols 3 to 5) isolated from root of the *P. emblica* L., showed moderate anti-TMV activity, with inhibition rates of 79.6 and 62.1% at a concentration of 1 mg/mL, respectively [33].

### 3.14. Miscellaneous

Candidiasis (oral thrush) is one of the frequent issues that affects good oral health. For centuries, *P. reticulatus* twigs have been used as chew sticks and the dried leaves powder also used to maintain oral health [125]. Its huge phytochemicals content makes the genus *Phyllanthus* an important and desirable group that could represent a tremendous business opportunity for developing countries. Almost each and every part of the plants of *Phyllanthus* species can be used due to the diverse chemistry of their compounds, and they cure various human ailments. A large number of commercial mouth hygiene products are made from the twigs, leaves, roots, fruits, fruit seeds, and flowers of *P. reticulates* [125]. 

*P. emblica* and *P. amarus* extracts are reported to be gastroprotective against gastric ulcers and gastritis by enhancing gastric motility [126,127].

Phyllanthin is present in almost all of the *Phyllanthus* species, and its supplementation is able to reduce mRNA expression of adipogenic genes and increase the expression of lipolytic genes in white adipose tissue. Moreover, phyllanthin increases the expression levels of mRNA of the insulin receptor and insulin receptor subtrate-1 in adipose and liver tissues, and hence provides protection against metabolic damage in tissues [20]. Obesity causes poor lifestyles by increasing health issues such as heart disease, type 2 diabetes, obstructive sleep apnea and cancers. The aqueous extracts of *Phyllanthus* play a vital role in the body and weight loss by significant improvement in serum glucose, liver enzymes, lipid profile, apolipoprotien A and kidney functions in afflicted obese rats [128]. A famous wine is being brewed from *P. emblica* and *P. acidus* from both wild as well as cultivated species, which shows increased antioxidant activities following fermentation [129]. It is noteworthy that cultivated *P. emblica* wine is sweeter than other wines.

## 4. Conclusions

*Phyllanthus* is one of the largest genus in the family Phyllanthaceae and is in constant use in traditional medications to cure diverse human diseases, in confectionaries, food industry, and also in some pesticides. *Phyllanthus* species enriched with diversity of phytochemicals e.g., tannins, terpenes, alkaloids, glycosidic compounds, saponins, and flavones etc. During 2016–2018, almost 81 compounds have been isolated from *Phyllanthus* spp. the majority of which are phenylpropanoids, triterpenoids, diterpenoids, and flavonoids. These chemical compounds from *Phyllanthus* species bear diverse biological activities, and hence demand indepth pharmacological studies for their potential use in pharma industry.

## Figures and Tables

**Table 1:** Structures and sources of phenylpropanoids from different *Phyllanthus species.*

No.	Compounds	Sources	Ref.
**1**	(+)-(7*R*,8*S*)-phyllanglaucin A	*P. glaucus*	[23]
**2**	(−)-(7*S*,8*R*)-phyllanglaucin A	*P. glaucus*	[23]
**3**	(7*R*,8*S*)-dihydrodehyd roconiferyl alcohol	*P. glaucus*	[23]
**4**	(7*S*,8*R*)-dihydrodehydrodiconiferyl alcohol	*P. glaucus*	[23]
**5**	(7*R*,8*R*)-4,4′-dihydroxy-3,7,3′-trimethoxy-8,1′-7′,8′,9′-trinor-neolignan-9-ol	*P. glaucus*	[23]
**6**	(7*S*,8*S*)-4,4′ -dihydroxy-3,7,3′-trimethoxy-8,1′-7′,8′,9′-trinor-neolignan-9-ol	*P. glaucus*	[23]
**7**	(7*S*,8*R*)-4,4′-dihydroxy-3,7,3′-trimethoxy-8,1′-7′,8′,9′-trinor-neolignan-9-ol	*P. glaucus*	[23]
**8**	(7*R*,8*S*)-4,4′-dihydroxy-3,7,3′-trimethoxy-8,1′-7′,8′,9′-trinor-neolignan-9-ol	*P. glaucus*	[23]
**9**	(+)-phyllanglaucin B	*P. glaucus*	[23]
**10**	(−)-phyllanglaucin B	*P. glaucus*	[23]
**11**	(+)-phyllanglaucin C	*P. glaucus*	[23]
**12**	(−)-phyllanglaucin C	*P. glaucus*	[23]
**13**	(+)-phyllanglaucin D	*P. glaucus*	[23]
**14**	(−)-phyllanglaucin D	*P. glaucus*	[23]
**15**	(7*R*,8*S*,7′*R*,8′*R*)-acernikol	*P. glaucus*	[23]
**16**	(7*S*,8*R*,7′*S*,8′*S*)-acernikol	*P. glaucus*	[23]
**17**	(7*R*,8*S*,7′*S*,8′*S*)-acernikol	*P. glaucus*	[23]
**18**	(7*S*,8*R*,7′*R*,8′*R*)-acernikol	*P. glaucus*	[23]
**19**	(7*S*,8*R*)-cedrusin	*P. glaucus*	[23]
**20**	(7*S*,8*R*)-dihydrodehydrodiconifenyl alcohol 9-*O*-β-d-xylopyranoside	*P. glaucus*	[23]
**21**	(7*S*,8*R*)-4,7,9,9′-tetrahydroxy-3,3′-dimethoxy-8-*O*-4′-neolignan	*P. glaucus*	[23]
**22**	5-demethoxy-niranthin	*P. amarus*	[25]
**23**	niranthin	*P. amarus*	[25,26]
**24**	phyllanthin	*P. amarus*	[25,26]
**25**	filtetralin	*P. amarus*	[25]
**26**	5-demethoxy-nirtetralin	*P. amarus*	[25]
**27**	nirtetralin	*P. amarus*	[25]
**28**	hipophyllanthin	*P. amarus*	[25]
**29**	cinnamic acid	*P. amarus*, *P. urinaria*	[24]
**30**	phenylalanine	*P. amarus*,*P. urinaria*	[24]
**31**	5-*O*-β-d-glucopyranosyljusticidin B	*P. brasiliensis*	[27]
**32**	justicidin B	*P. brasiliensis*	[27]
**33**	cleistanthin B	*P. brasiliensis*	[27]
**34**	arabelline	*P. brasiliensis*	[27]
**35**	4-*O*-β-d-apiofuranosyl-(1′″→6′′)-β-d-glucopyranosyldiphyllin	*P. brasiliensis*	[27]
**36**	tuberculatin	*P. brasiliensis*	[27]
**37**	phyllanthostatin A	*P. brasiliensis*	[27]

**Table 2:** Structures and sources of terpenoids from various *Phyllanthus* species.

No.	Compounds	Sources	Ref.
**38**	phainanolide A	*P. hainanensis*	[28]
**39**	phainanoid G	*P. hainanensis*	[28]
**40**	phainanoid H	*P. hainanensis*	[28]
**41**	phainanoid I	*P. hainanensis*	[28]
**42**	28-norlup-20(29)-ene-3,17β-diol	*P. urinaria*	[29]
**43**	betulin	*P. urinaria*	[29]
**44**	β-betulinic acid	*P. urinaria*	[29]
**45**	3-oxofriedelan-28-oic acid	*P. urinaria*	[29]
**46**	oleanolic acid	*P. urinaria*	[29]
**47**	(*E*)-coumaroyltaraxerol	*P. urinaria*	[29]
**48**	(*Z*)-coumaroyltaraxerol	*P. urinaria*	[29]
**49**	phyllaciduloid A	*P. acidus*	[32]
**50**	spruceanol	*P. acidus*	[31]
**51**	phyllaciduloid B	*P. acidus*	[32]
**52**	phyllaciduloid C	*P. acidus*	[32]
**53**	phyllaciduloid D	*P. acidus*	[32]
**54**	phyllanes A	*P. acidus*	[31]
**55**	phyllanes B	*P. acidus*	[31]
**56**	(−)-loliolide	*P. urinaria*	[30]

## References

[1] Haque T., Muhsin M.D.A., Akhter T., Haq M.E., Begum R., Chowdhury S.F.U.A. (2016). Antimicrobial and analgesic activity of leaf extracts of *Phyllanthus reticulatus* Poir. (Family-Euphorbiaceae). Jahangirnagar Univ. J. Biol. Sci..

[2] Petrovska B.B. (2012). Historical review of medicinal plants’ usage. Pharmacogn. Rev..

[3] Aboelsoud N.H. (2010). Herbal medicine in ancient Egypt. J. Med. Plants Res..

[4] Gismondi A., D’Agostino A., Canuti L., Di Marco G., Martínez-Labarga C., Angle M., Rickard O., Canini A. (2018). Dental calculus reveals diet habits and medicinal plant use in the Early Medieval Italian population of Colonna. J. Archaeol. Sci. Rep..

[5] Manjula V., Norman T.S.J. (2017). *Phyllanthus reticulatus* for oral health. J. Med. Plants.

[6] De Oliveira C.N.F., Frezza T.F., Garcia V.L., Figueira G.M., Mendes T.M.F., Allegretti S.M. (2017). *Schistosoma mansoni*: In vivo evaluation of *Phyllanthus amarus* hexanic and ethanolic extracts. Exp. Parasitol..

[7] Mao X., Wu L.F., Guo H.L., Chen W.J., Cui Y.P., Qi Q., Li S., Liang W.Y., Yang G.H., Shao Y.Y. (2016). The genus *Phyllanthus*: An ethnopharmacological, phytochemical, and pharmacological review. Evid. Based Complement. Altern..

[8] Devi S., Kumar D., Kumar M. (2016). In-Vitro antioxidant activities of methanolic extract of whole plant of *Phyllanthus amarus* (Euphorbiaceae). Int. J. Bot. Stud..

[9] Devi S., Kumar M. (2017). In vitro Antioxidant potential of methanolic extract of whole plant of *Phyllanthus amarus* Schum (Euphorbiaceae). Int. J. Bot. Stud..

[10] Kaur B., Kaur N., Gautam V. (2016). Evaluation of anti-helicobacter pylori (DSMZ 10242) activity and qualitative analysis of quercetin by HPLC in *Phyllanthus niruri* linn. World J. Pharm. Pharm. Sci..

[11] Nguyen V.T., Pham H.N.T., Bowyer M.C., van Altena I.A., Scarlett C.J. (2016). Influence of solvents and novel extraction methods on bioactive compounds and antioxidant capacity of *Phyllanthus amarus*. Chem. Pap..

[12] Zhang J., Miao D., Zhu W.F., Xu J., Liu W.Y., Kitdamrongtham W., Manosroi J., Abe M., Akihisa T., Feng F. (2017). Biological activities of phenolics from the fruits of *Phyllanthus emblica* L. (Euphorbiaceae). Chem. Biodivers..

[13] Tung Y.-T., Huang C.-Z., Lin J.-H., Yen G.-C. (2018). Effect of *Phyllanthus emblica* L. fruit on methionine and choline-deficiency diet-induced nonalcoholic steatohepatitis. J. Food Drug Anal..

[14] Boakye-Gyasi E., Kasange E.A., Biney R.P., Boadu-Mensah K., Agyare C., Woode E. (2016). Anti-nociceptive effects of geraniin and an aqueous extract of the aerial parts of *Phyllanthus muellerianus* (Kuntze) Exell. in murine models of chemical nociception. Iran. J. Pharm. Sci..

[15] Mostofa R., Ahmed S., Begum M.M., Sohanur Rahman M., Begum T., Ahmed S.U., Tuhin R.H., Das M., Hossain A., Sharma M. (2017). Evaluation of anti-inflammatory and gastric anti-ulcer activity of *Phyllanthus niruri* L. (Euphorbiaceae) leaves in experimental rats. BMC Complement. Altern. Med..

[16] Menéndez-Perdomo I.M., Wong-Guerra M., Fuentes-León F., Carrazana E., Casadelvalle I., Vidal A., Sánchez-Lamar Á. (2017). Antioxidant, photoprotective and antimutagenic properties of *Phyllanthus* spp. from Cuban flora. J. Pharm. Pharmacogn. Res..

[17] Guo X., Wang X. (2016). *Phyllanthus emblica* Fruit Extract Activates Spindle Assembly Checkpoint, Prevents Mitotic Aberrations and Genomic Instability in Human Colon Epithelial NCM460 Cells. Int. J. Mol. Sci..

[18] Noorudheen N., Chandrasekharan D.K. (2016). Effect of ethanolic extract of *Phyllanthus emblica* on captan induced oxidative stress in vivo. South Indian J. Biol. Sci..

[19] Guo X.H., Ni J., Xue J.L., Wang X. (2017). *Phyllanthus emblica* Linn. fruit extract potentiates the anticancer efficacy of mitomycin C and cisplatin and reduces their genotoxicity to normal cells in vitro. J. Zhejiang Univ. Sci. B.

[20] Jagtap S., Khare P., Mangal P., Kondepudi K.K., Bishnoi M., Bhutani K.K. (2016). Protective effects of phyllanthin, a lignan from *Phyllanthus amarus*, against progression of high fat diet induced metabolic disturbances in mice. RSC Adv..

[21] Lee N.Y., Khoo W.K., Adnan M.A., Mahalingam T.P., Fernandez A.R., Jeevaratnam K. (2016). The pharmacological potential of *Phyllanthus niruri*. J. Pharm. Pharmacol..

[22] Yang B., Kortesniemi M., Liu P., Karonen M., Salminen J.P. (2012). Analysis of hydrolyzable tannins and other phenolic compounds in emblic leafflower (*Phyllanthus emblica* L.) fruits by high performance liquid chromatography–electrospray ionization mass spectrometry. J. Agric. Food Chem..

[23] Wu Z., Lai Y., Zhou L., Wu Y., Zhu H., Hu Z., Yang J., Zhang J., Wang J., Luo Z. (2016). Enantiomeric lignans and neolignans from *Phyllanthus glaucus*: Enantioseparation and their absolute configurations. Sci. Rep..

[24] Muthusamy A., Prasad H.N.N., Sanjay E.R., Rao M.R., Satyamoorthy K. (2016). Impact of precursors and plant growth regulators on in vitro growth, bioactive lignans, and antioxidant content of *Phyllanthus* species. In Vitro Cell. Dev. Plant.

[25] Pereira R.G., Garcia V.L., Rodrigues M.V.N., Martínez J. (2016). Extraction of lignans from *Phyllanthus amarus* Schum. & Thonn using pressurized liquids and low pressure methods. Sep. Purif. Technol..

[26] Pereira R.G., Nakamura R.N., Rodrigues M.V.N., Osorio-Tobón J.F., Garcia V.L., Martinez J. (2017). Supercritical fluid extraction of phyllanthin and niranthin from *Phyllanthus amarus* Schum. & Thonn. J. Supercrit. Fluids.

[27] Borges L.D.C., Negrão-Neto R., Pamplona S., Fernandes L., Barros M., Fontes-Júnior E., Maia C., Silva C.Y.Y., Silva M.N.D. (2018). Anti-Inflammatory and Antinociceptive Studies of Hydroalcoholic Extract from the Leaves of *Phyllanthus brasiliensis* (Aubl.) Poir. and Isolation of 5-*O*-β-d-Glucopyranosyljusticidin B and Six Other Lignans. Molecules.

[28] Fan Y.Y., Gan L.S., Liu H.C., Li H., Xu C.H., Zuo J.P., Ding J., Yue J.M., Phainanolide A. (2017). Highly modified and oxygenated triterpenoid from *Phyllanthus hainanensis*. Org. Lett..

[29] Wu Y., Xie S.S., Hu Z.X., Wu Z.D., Guo Y., Zhang J.W., Wang J.P., Xue Y.B. (2017). Triterpenoids from Whole Plants of *Phyllanthus urinaria*. Chin. Herb. Med..

[30] Chung C.Y., Liu C.H., Burnouf T., Wang G.H., Chang S.P., Jassey A., Tai C.J., Tai C.J., Huang C.J., Richardson C.D. (2016). Activity-based and fraction-guided analysis of *Phyllanthus urinaria* identifies loliolide as a potent inhibitor of hepatitis C. virus entry. Antivir. Res..

[31] Duong T.H., Bui X.H., Pogam P.L., Nguyen H.H., Tran T.T., Nguyen T.A.T., Chavasiri W., Boustie J., Nguyen K.P.P. (2017). Two novel diterpenes from the roots of *Phyllanthus acidus* (L.) Skeel. Tetrahedron.

[32] Zheng X.H., Yang J., Lv J.J., Zhu H.T., Wang D., Xu M., Yang C.R., Zhang Y.J. (2018). Phyllaciduloids A-D: Four new cleistanthane diterpenoids from *Phyllanthus acidus* (L.) Skeels. Fitoterapia.

[33] Yan H., Han L.R., Zhang X., Feng J.T. (2017). Two new Anti-TMV active chalconoid analogues from the root of *Phyllanthus emblica*. Nat. Prod. Res..

[34] Zhao J., Liu T.T., Chen G. (2016). An effective β-cyclodextrin polyurethane spherical adsorbent for the chromatographic enrichment of corilagin from *Phyllanthus niruri* L. extract. React. Funct. Polym..

[35] Tram N.C.T., Son N.T., Thao D.T., Cuong N.M. (2016). Kaempferol and kaempferol glycosides from *Phyllanthus acidus* leaves. Vietnam J. Chem..

[36] Putakala M., Gujjala S., Nukala S., Bongu S.B.R., Chintakunta N., Desireddy S. (2017). Cardioprotective effect of *Phyllanthus amarus* against high fructose diet induced myocardial and aortic stress in rat model. Biomed. Pharmacother..

[37] Komlaga G., Genta-Jouve G., Cojean S., Dickson R.A., Mensah M.L., Loiseau P.M., Champy P., Beniddir M.A. (2017). Antiplasmodial Securinega alkaloids from *Phyllanthus fraternus*: Discovery of natural (+)-allonorsecurinine. Tetrahedron Lett..

[38] Zeng Z., Lv W., Jing Y., Chen Z., Song L., Liu T., Yu R. (2017). Structural characterization and biological activities of a novel polysaccharide from *Phyllanthus emblica*. Drug Discov. Ther..

[39] Navarro M., Moreira I., Arnaez E., Quesada S., Azofeifa G., Alvarado D., Monagas M.J. (2017). Proanthocyanidin Characterization, Antioxidant and Cytotoxic Activities of Three Plants Commonly Used in Traditional Medicine in Costa Rica: *Petiveria alliaceae* L., *Phyllanthus niruri* L. and *Senna reticulata* Willd. Plants.

[40] Navarro M., Moreira I., Arnaez E., Quesada S., Azofeifa G., Vargas F., Alvarado D., Chen P. (2017). Flavonoids and Ellagitannins Characterization, Antioxidant and Cytotoxic Activities of *Phyllanthus acuminatus* Vahl. Plants.

[41] Laulloo S.J., Bhowon M., Chua L., Gaungoo H. (2018). Phytochemical Screening and Antioxidant Properties of *Phyllanthus emblica* from Mauritius. Chem. Nat. Compd..

[42] Kumar S., Singh A., Bajpai V., Singh B., Kumar B. (2017). Development of a UHPLC–MS/MS method for the quantitation of bioactive compounds in *Phyllanthus* species and its herbal formulations. J. Sep. Sci..

[43] Kumar S., Singh A., Kumar B. (2017). Identification and characterization of phenolics and terpenoids from ethanolic extracts of *Phyllanthus* species by HPLC-ESI-QTOF-MS/MS. J. Pharm. Anal..

[44] Ghafar S.Z.A., Mediani A., Ramli N.S., Abas F. (2018). Antioxidant, α-glucosidase, and nitric oxide inhibitory activities of *Phyllanthus acidus* and LC–MS/MS profile of the active extract. Food Biosci..

[45] Ojezele M.O., Moke E.G., Onyesom I. (2017). Impact of generic antimalarial or *Phyllanthus amarus* and vitamin co-administration on antioxidant status of experimental mice infested with *Plasmodium berghei*. Beni-Suef Univ. J. Basic Appl. Sci..

[46] Peter S., Dey S., Veerakyathappa B., Kumar S.R., Paulad C. (2016). Therapeutic activity of Partially purified fractions of *Emblica officinalis* (Syn. *Phyllanthus emblica*) dried fruits against *Trypanosoma evansi*. J. Pharm. Pharmacol..

[47] Chansriniyom C., Bunwatcharaphansakun P., Eaknai W., Nalinratana N., Ratanawong A., Khongkow M., Luechapudiporn R. (2018). A synergistic combination of *Phyllanthus emblica* and Alpinia galanga against H_2_O_2_ -induced oxidative stress and lipid peroxidation in human ECV304 cells. J. Funct. Foods.

[48] Akporowhe S., Onyesom I. (2016). *Phyllanthus amarus* augments the serum antioxidant capacity and invigorates the blood in experimental mice. Biosci. Biotechnol. Res. Commun..

[49] Rusmana D., Wahyudianingsih R., Elisabeth M., Balqis B., Maesaroh M., Widowati W. (2017). Antioxidant Activity of *Phyllanthus niruri* Extract, Rutin and Quercetin. Indones. Biomed. J..

[50] Singh R.P., Pal A., Pal K. (2016). Antioxidant activity of ethanolic and aqueous extract of *Phyllanthus niruri*—In vitro. World J. Pharm. Pharm. Sci..

[51] Angamuthu J., Ganapathy M., Evanjelene V.K. (2016). Evaluation of antioxidant activity of *Phyllanthus acidus*. World J. Pharm. Pharm. Sci..

[52] Nguyen V.T., Sakoff J.A., Scarlett C.J. (2017). Physicochemical Properties, Antioxidant and Cytotoxic Activities of Crude Extracts and Fractions from *Phyllanthus amarus*. Medicines.

[53] Ramandeep K., Nahid A., Neelabh C., Navneet K. (2017). Phytochemical Screening of *Phyllanthus niruri* collected from Kerala Region and its Antioxidant and Antimicrobial Potentials. J. Pharm. Sci. Res..

[54] Dai M., Wahyuni A., Dk I., Azizah T., Suhendi A., Saifudin A. (2016). Antioxidant activity of *Phyllanthus niruri* L. herbs: In vitro and in vivo models and isolation of active compound. Nat. J. Physiol. Pharm. Pharmacol..

[55] Andrianto D., Widianti W., Bintang M. (2017). Antioxidant and Cytotoxic Activity of *Phyllanthus acidus* Fruit Extracts. IOP Conf. Ser. Earth Environ. Sci..

[56] Zhang Y., Zhao L., Guo X., Li C., Li H., Lou H., Ren D. (2014). Chemical constituents from *Phyllanthus emblica* and the cytoprotective effects on H_2_O_2_-induced PC12 cell injuries. Arch. Pharm. Res..

[57] Kalpana S., Ramakrushna B., Anitha S. (2016). Evaluation of in vitro antioxidant and α-amylase inhibitory activity of *Phyllanthus indofischeri* Bennet. Int. J. Pharm. Pharm. Sci..

[58] Sabir S.M., Shah R.H., Shah A.H. (2017). Total Phenolic and Ascorbic Acid Contents and Antioxidant Activities of Twelve Different Ecotypes of *Phyllanthus emblica* from Pakistan. Chiang Mai J. Sci..

[59] Tahir I., Khan M.R., Shah N.A., Aftab M. (2016). Evaluation of phytochemicals, antioxidant activity and amelioration of pulmonary fibrosis with *Phyllanthus emblica* leaves. BMC Complement. Altern. Med..

[60] Boakye Y.D., Agyare C., Dapaah S.O. (2016). In vitro and in vivo antioxidant properties of *Phyllanthus muellerianus* and its major constituent, geraniin. Oxid. Antioxid. Med. Sci..

[61] Huang S.T., Huang C.C., Sheen J.M., Lin T.K., Liao P.L., Huang W.L., Wang P.W., Liou C.W., Chuang J.H. (2016). *Phyllanthus urinaria*’s Inhibition of Human Osteosarcoma Xenografts Growth in Mice is Associated with Modulation of Mitochondrial Fission/Fusion Machinery. Am. J. Chin. Med..

[62] Perera D., Soysa P., Wijeratne S. (2016). Polyphenols contribute to the antioxidant and antiproliferative activity of *Phyllanthus debilis* plant in-vitro. BMC Complement. Altern. Med..

[63] Zheng Z.Z., Chen L.H., Liu S.S., Deng Y., Zheng G.H., Gu Y., Ming Y.L. (2016). Bioguided Fraction and Isolation of the Antitumor Components from *Phyllanthus niruri* L.. Biomed. Res. Int..

[64] Guo J.R., Chen Q.Q., Lam C.W., Wang C.Y., Xu F.G., Liu B.M., Zhang W. (2016). Effect of *Phyllanthus amarus* Extract on 5-Fluorouracil-Induced Perturbations in Ribonucleotide and Deoxyribonucleotide Pools in HepG2 Cell Line. Molecules.

[65] Sawitri E. (2016). Apoptosis of Colorectal Cancer Cell on Sprague-Dawley Rats Induced with 1, 2 Dimethylhidrazine and *Phyllanthus niruri* Linn Extrac. Int. J. Sci. Eng..

[66] Wang C.C., Yuan J.R., Wang C.F., Yang N., Chen J., Liu D., Song J., Feng L., Tan X.B., Jia X.B. (2017). Anti-inflammatory Effects of *Phyllanthus emblica* L. on Benzopyrene-Induced Precancerous Lung Lesion by Regulating the IL-1beta/miR-101/Lin28B Signaling Pathway. Integr. Cancer Ther..

[67] Wang F., Wang H. (2017). *Phyllanthus emblica* L. extract activates Nrf2 signalling pathway in HepG2 cells. Biomed. Res..

[68] Desai K., Braganza V. (2016). Cytotoxic activity and phytochemical investigation of *Phyllanthus emblica* L. leaves. Int. J. Pharm. Sci. Res..

[69] Stefanowicz-Hajduk J., Sparzak-Stefanowska B., Krauze-Baranowska M., Ochocka J.R. (2016). Securinine from *Phyllanthus glaucus* Induces Cell Cycle Arrest and Apoptosis in Human Cervical Cancer HeLa Cells. PLoS ONE.

[70] Vernhes Tamayo M., Schuch A.P., Yagura T., Baly Gil L., Menck C.F.M., Sánchez-Lamar A. (2018). Genoprotective effect of *Phyllanthus orbicularis* extract against UVA, UVB and solar radiation. Photochem. Photobiol..

[71] Tansirikongkol A. (2016). Comparative in vitro anti-aging activities of *Phyllanthus emblica* L. extract, *Manilkara sapota* L. extract and its combination. Thai J. Pharm. Sci..

[72] Srinivasan P., Vijayakumar S., Kothandaraman S., Palani M. (2018). Anti-diabetic activity of quercetin extracted from *Phyllanthus emblica* L. fruit: In silico and in vivo approaches. J. Pharm. Anal..

[73] Rao N.K., Bethala K., Sisinthy S.P., Manickam S. (2016). Antihyperglycemic and In Vivo Antioxidant Activities of *Phyllanthus watsonii* A. Shaw Roots in Streptozotocin Induced Type 2 Diabetic Rats. Int. J. Pharmacogn. Phytochem. Res..

[74] Singh S., Chauhan M.G., Kaur B., Kumar B., Gulati M., Singh S.K. (2017). Characterization, organoleptic evaluation and standardization of aqueous extracts of antidiabetic herbs *Trigonella foenum*, *Allium sativum*, *Aloe vera*, *Phyllanthus niruri*. J. Pharm. Res..

[75] Pathak N., Bandyopadhyay A., Kumar G., Chaurasia R., Varma K. (2016). Comparative study to evaluate the anti-diabetic activity of commercially available extract of *Tinospora cordifolia* and *Phyllanthus emblica* in streptozocin induced diabetic rat. Int. J. Basic Clin. Pharmacol..

[76] Zhou J., Zhang C., Zheng G.H., Qiu Z. (2018). Emblic Leaf flower (*Phyllanthus emblica* L.) Fruits Ameliorate Vascular Smooth Muscle Cell Dysfunction in Hyperglycemia: An Underlying Mechanism Involved in Ellagitannin Metabolite Urolithin A. Evid. Based Complement. Altern. Med..

[77] Nadro M.S., Elkanah G. (2017). Hypoglycaemic effect of fractions and crude methanolic leaf extract of *Phyllanthus fraternus* in streptozotocin—Induced diabetic and normal rats. J. Med. Plants Res..

[78] Beidokhti M.N., Andersen M.V., Eid H.M., Villavicencio M.L.S., Staerk D., Haddad P.S., Jäger A.K. (2017). Investigation of antidiabetic potential of *Phyllanthus niruri* L. using assays for α-glucosidase, muscle glucose transport, liver glucose production, and adipogenesis. Biochem. Bioph. Res. Commun..

[79] Mediani A., Abas F., Maulidiani M., Khatib A., Tan C.P., Ismail I.S., Shaari K., Ismail A., Lajis N.H. (2016). Metabolic and biochemical changes in streptozotocin induced obese-diabetic rats treated with *Phyllanthus niruri* extract. J. Pharm. Biomed. Anal..

[80] Hossain M.N., Rahmatullah M. (2016). Oral glucose tolerance tests with a formulation containing *Phyllanthus emblica* fruits and *Trigonella foenum-graecum* seeds. World J. Pharm. Pharm. Sci..

[81] Bongu S.B.R., Sagree S., Gudapareddy V., Putakala M., Nukala S., Gujjala S., Bellamkonda R., Desireddy S. (2016). Protective role of aqueous extract of *Phyllanthus amarus* on oxidative stress in pancreas of streptozotocin induced diabetic male Wistar rats. J. Exp. Appl. Anim. Sci..

[82] Chaimum-aom N., Chomko S., Talubmook C. (2016). Toxicology and Oral glucose Tolerance Test (OGTT) of Thai Medicinal Plant Used for Diabetes controls, *Phyllanthus acidus* L. (Euphorbiaceae). Pharmacogn. J..

[83] Olubunmi O.P., Yinka O.S., Oladele O.J., John O.A., Boluwatife B.D., Oluseyi F.S. (2017). Aberrations in Renal Function Parameters Following Oral Administration of *Phyllanthus amarus* in Cadmium-Induced Kidney Damage in Adult Wistar Rats. J. Dis. Med. Plants.

[84] Abbas N., Naz M., Alyousef L., Ahmed E.S., Begum A. (2017). Comparative study of hepatoprotective effect produced by *Cuminum cyminum*, fruits of *Phyllanthus emblicus* and silymarin against cisplatin-induced hepatotoxicity. Int. J. Pharm. Sci. Res..

[85] Chaphalkar R., Apte K.G., Talekar Y., Ojha S.K., Nandave M. (2017). Antioxidants of *Phyllanthus emblica* L. bark extract provide hepatoprotection against ethanol-induced hepatic damage: A comparison with silymarin. Oxid. Med. Cell. Longev..

[86] Iqbal Z., Asif M., Aslam N., Akhtar N., Asmawi M.Z., Fei Y.M., Jabeen Q. (2017). Clinical investigations on gastroprotective effects of ethanolic extract of *Phyllanthus emblica* L. fruits. J. Herb. Med..

[87] Hassan M.R.A., Mustapha N.R.N., Jaya F., Arjunan S., Ooi E.T., Said R.M., Menon J., Tee H.P., Omar H., Aiman S. (2017). Efficacy and Safety of *Phyllanthus niruri* in Non-alcoholic Steatohepatitis Treatment: Pilot Study from Malaysia. J. Pharm. Pract. Commun. Med..

[88] Lu C.C., Yang S.H., Hsia S.M., Wu C.H., Yen G.C. (2016). Inhibitory effects of *Phyllanthus emblica* L. on hepatic steatosis and liver fibrosis in vitro. J. Funct. Foods.

[89] Yao N., Kamagaté M., Amonkan A.K., Koffi C., Kpahe F., Kouamé M., Dié-Kacou H. (2016). Comparative effects of aqueuos extract of *Phyllanthus amarus* and its fractions on urinary excretion in rat. J. Phytopharmacol..

[90] Yao A.N., Kamagate M., Amonkan A.K., Chabert P., Kpahe F., Koffi C., Kouame M.N., Auger C., Kati-Coulibaly S., Schini-Kerth V. (2018). The acute diuretic effect of an ethanolic fraction of *Phyllanthus amarus* (Euphorbiaceae) in rats involves prostaglandins. BMC Complement. Altern. Med..

[91] Uddin M.S., Mamun A.A., Hossain M.S., Akter F., Iqbal M.A., Asaduzzaman M. (2016). Exploring the Effect of *Phyllanthus emblica* L. on Cognitive Performance, Brain Antioxidant Markers and Acetylcholinesterase Activity in Rats: Promising Natural Gift for the Mitigation of Alzheimer’s Disease. Ann. Neurosci..

[92] Uddin M.S., Mamun A.A., Hossain M.S., Ashaduzzaman M., Noor M.A.A., Hossain M.S., Uddin M.J., Sarker J., Asaduzzaman M. (2016). Neuroprotective Effect of *Phyllanthus acidus* L. on Learning and Memory Impairment in Scopolamine-Induced Animal Model of Dementia and Oxidative Stress: Natural Wonder for Regulating the Development and Progression of Alzheimer’s Disease. Adv. Alzheimer’s Dis..

[93] Jang H., Srichayet P., Park W.J., Heo H.J., Kim D.-O., Tongchitpakdee S., Kim T.-J., Jung S.H., Lee C.Y. (2017). *Phyllanthus emblica* L. (Indian gooseberry) extracts protect against retinal degeneration in a mouse model of amyloid beta-induced Alzheimer’s disease. J. Funct. Foods.

[94] Wagle N., Nagarjuna S., Sharma H., Dangi N.B., Sapkota H.P., Naik B.S., Padhaya R.R. (2016). Evaluation of antinociceptive and anti-inflammatory activity of phytosterol present in chloroform extract of *Phyllanthus maderaspatensis*. Indian J. Physiol. Pharmacol..

[95] Hossain M., Akter S., Begum Y., Bulbul I. (2016). Analgesic and Anti-inflammatory Activities of Ethanolic Leaf Extract of *Phyllanthus acidus* L. on Swiss Albino Mice. Eur. J. Med. Plants.

[96] Yoon W.H., Lee K.H. (2017). Anti-inflammatory, Anti-arthritic and Analgesic Effect of the Herbal Extract Made from *Bacopa monnieriis*, *Cassia fistula* and *Phyllanthus polyphyllus*. Nat. Prod. Sci..

[97] Manikandan R., Beulaja M., Thiagarajan R., Palanisamy S., Goutham G., Koodalingam A., Prabhu N.M., Kannapiran E., Basu M.J., Arulvasu C. (2017). Biosynthesis of silver nanoparticles using aqueous extract of *Phyllanthus acidus* L. fruits and characterization of its anti-inflammatory effect against H_2_O_2_ exposed rat peritoneal macrophages. Process Biochem..

[98] Hossain M., Akter S., Das A., Sarwar M. (2016). CNS Depressant, Antidiarrheal and Antipyretic Activities of Ethanolic Leaf Extract of *Phyllanthus acidus* L. on Swiss Albino Mice. Br. J. Pharm. Res..

[99] Khan A., Ahmed T., Rizwan M., Khan N. (2018). Comparative therapeutic efficacy of *Phyllanthus emblica* (Amla) fruit extract and procaine penicillin in the treatment of subclinical mastitis in dairy buffaloes. Microb. Pathog..

[100] Afrin F., Banik S., Hossain M.S. (2016). Pharmacological activities of methanol extract of *Phyllanthus acidus* pulp. J. Med. Plants Res..

[101] Tjandrawinata R.R., Susanto L.W., Nofiarny D. (2017). The use of *Phyllanthus niruri* L. as an immunomodulator for the treatment of infectious diseases in clinical settings. Asian Pac. J. Trop. Dis..

[102] Putri D.U., Rintiswati N., Soesatyo M.H., Haryana S.M. (2018). Immune modulation properties of herbal plant leaves: *Phyllanthus niruri* aqueous extract on immune cells of tuberculosis patient—In vitro study. Nat. Prod. Res..

[103] Muthulakshmi M., Subramani P., Michael R. (2016). Immunostimulatory effect of the aqueous leaf extract of *Phyllanthus niruri* on the specific and nonspecific immune responses of *Oreochromis mossambicus* Peters. Iran. J. Vet. Res..

[104] Ilangkovan M., Jantan I., Bukhari S.N. (2016). Phyllanthin from *Phyllanthus amarus* inhibits cellular and humoral immune responses in Balb/C mice. Phytomedicine.

[105] Ilangkovan M., Jantan I., Mesaik M.A., Bukhari S.N. (2016). Inhibitory Effects of the Standardized Extract of *Phyllanthus amarus* on Cellular and Humoral Immune Responses in Balb/C Mice. Phytother. Res..

[106] Sabdoningrum E.K., Hidanah S., Wahjuni R.S., Chusniati S., Arimbi A. (2017). An in vitro antibacterial activity test of Meniran Herbs’ (*Phyllanthus Niruri* L.) ethanol extract against *Mycoplasma gallisepticum* causes Chronic Respiratory Disease (CRD) in Broiler Chickens. KnE Life Sci..

[107] Senjobi C.T., Ettu A.O., Otujo C.O. (2017). Antibacterial and antifungal activities of leaf extracts of *Phyllanthus amaru* Schum and Thonn. J. Pharmacogn. Phytother..

[108] Boakye Y.D., Agyare C., Hensel A. (2016). Anti-infective properties and time-kill kinetics of Phyllanthus muellerianus and its major constituent, geraniin. Med. Chem..

[109] Adesegun A., Samuel F., Adesina O. (2016). Antibacterial Activity of the Volatile Oil of *Phyllanthus muellerianus* and Its Inhibition against the Extracellular Protease of Klebsiella granulomatis. Eur. J. Med. Plants.

[110] Pathmavathi M., Thamizhiniyan P. (2016). Antimicrobial activity of various extracts of *Plectranthus ambionicus* and *Phyllanthus amarus*. J. Appl. Adv. Res..

[111] Sathishkumar M., Saroja M., Venkatachalam M., Rajamanickam A. (2017). Biosynthesis of zinc sulphide nanoparticles using *Phyllanthus emblica* and their antimicrobial activities. Elixir Elec. Eng..

[112] Oluboyo B.O., Oluboyo A.O., Kalu S.O. (2016). Inhibitory effects of *Phyllanthus amarus* extracts on the growth of some pathogenic microorganisms. Afr. J. Clin. Exp. Microbiol..

[113] Gunawan I., Bawa I., Putra A.B. (2016). Isolation, characterization and antibacterial activity of triterpenoid compounds fraction chloroform bark *Phyllanthus niruri* L.. World Pharm. Pharm. Sci..

[114] Haris M., Kumar A., Ahmad A., Abuzinadah M.F., Basheikh M., Khan S.A., Mujeeb M. (2017). Microwave-assisted green synthesis and antimicrobial activity of silver nanoparticles derived from a supercritical carbon dioxide extract of the fresh aerial parts of *Phyllanthus niruri* L.. Trop. J. Pharm. Res..

[115] Uzor B., Umeh L., Manu O. (2016). Phytochemical Composition and Antimicrobial Potential of *Phyllanthus amarus* Leaf Extract Against Some Clinical Isolates. Niger. J. Microbiol..

[116] Ahamath J.M., Vahith R.A., Manivel V., Elamparithi R. (2017). Phytochemical Screening and Antimicrobial Activity of *Phyllanthus niruri*. J. Adv. Appl. Sci. Res..

[117] Gao Q., Li X., Huang H., Guan Y., Mi Q., Yao J. (2018). The Efficacy of a Chewing Gum Containing *Phyllanthus emblica* Fruit Extract in Improving Oral Health. Curr. Microbiol..

[118] Li Y., Li X., Wang J.-K., Kuang Y., Qi M.-X. (2017). Anti-hepatitis B viral activity of *Phyllanthus niruri* L. (Phyllanthaceae) in HepG2/C3A and SK-HEP-1 cells. Trop. J. Pharm. Res..

[119] Chitra R., Vadivel E., Rajamani K. (2016). Estimation of anti-hepatic viral compounds in *Phyllanthus amarus* in vitro cultures. J. Hortic. Sci..

[120] Sarma K., Borkakoty B., Parida P., Jakharia A., Dey D., Biswas D., Panda D., K Modi M., K Mohapatra P., Mahanta J. (2016). In Silico Identification of Natural Lead Molecules from the Genus of *Phyllanthus* Against Hepatitis B Virus Reverse Transcriptase. Nat. Prod. J..

[121] Faeji C.O., Oladunmoye M.K., Adebayo I.A., Adebolu T.T. (2017). In-ovo biological activities of *Phyllanthus amarus* leaf extracts against Newcastle disease virus. J. Med. Plants Res..

[122] Dinesh S., Sudharsana S., Mohanapriya A., Itami T., Sudhakaran R. (2017). Molecular docking and simulation studies of *Phyllanthus amarus* phytocompounds against structural and nucleocapsid proteins of white spot syndrome virus. 3 Biotech.

[123] Sundaram D., Kesavan K., Kumaravel H., Mohammed R.F., Tohru M., Toshiaki I., Raja S. (2016). Protective efficacy of active compounds from *Phyllanthus amarus* against white spot syndrome virus in freshwater crab (Paratelphusa hydrodomous). Aquac. Res..

[124] Zhang X., Xia Q., Yang G., Zhu D., Shao Y., Zhang J., Cui Y., Wang R., Zhang L. (2017). The anti-HIV-1 activity of polyphenols from *Phyllanthus urinaria* and the pharmacokinetics and tissue distribution of its marker compound, gallic acid. J. Tradit. Chin. Med. Sci..

[125] Manjula V., Norman T.S.J. (2017). Pharmacognostical study of *Phyllanthus reticulatus*: A tribal drug. Pharma Innov. J..

[126] Berezi E., Uwakwe A., Monago-Ighorodje C., Nwauche K. (2017). Gastroprotective potentials of aqueous leaf extracts of *Phyllanthus amarus* on ibuprofen-induced ulcer in Wistar rats. Int. J. Adv. Res. Biol. Sci..

[127] Joshi S., Gajbhiye S.V., Thatte U. (2016). Evaluation of gastric motility of *Phyllanthus emblica* and *Asparagus racemosus* in cold stress induced gastric damage. Int. J. Basic Clin. Pharmacol..

[128] Ahmed A.H. (2017). The Effect of Water Extracts of *Phyllanthus emblica* and *Costus speciousus* on Reducing Obesity in Albino Rats. Alex. Sci. Exch..

[129] Nambiar S.S., Venugopal K.S., Shetty N.P., Appaiah K.A. (2016). Fermentation induced changes in bioactive properties of wine from *Phyllanthus* with respect to atherosclerosis. J. Food Sci. Technol..

